# Supplementary material for: Aqueous amine enables sustainable monosaccharide, monophenol, and pyridine base coproduction in lignocellulosic biorefineries
Source: Nat Commun. 2024 Jan 25;15:734. doi: 10.1038/s41467-024-45073-w (PMC10810809; doi:10.1038/s41467-024-45073-w)
Supplement: Supplementary file 1 — Supplementary information [file 41467_2024_45073_MOESM1_ESM.pdf]

Supplementary Information

Aqueous amine enables sustainable monosaccharide, monophenol, and pyridine base coproduction in lignocellulosic biorefineries

Li Xu[1], Meifang Cao[1], Jiefeng Zhou[1], Yuxia Pang[1], Zhixian Li[1], Dongjie Yang[1], Shao-Yuan Leu[2], Hongming Lou[1,*], Xuejun Pan[3], Xueqing Qiu[4,*]

[1] *Guangdong Provincial Key Lab of Green Chemical Product Technology, State Key Laboratory of Pulp and Paper Engineering, School of Chemistry and Chemical Engineering, South China University of Technology, Guangzhou 510641, China.*

[2] *Department of Civil and Environmental Engineering, The Hong Kong Polytechnic University, Hong Kong.*

[3] *Department of Biological Systems Engineering, University of Wisconsin-Madison, Madison, WI 53706, USA.*

[4] *School of Chemical Engineering and Light Industry, Guangdong University of Technology, Guangzhou 510006, China.*

[*]Corresponding author.

E-mail address: cehmlou@scut.edu.cn (H. Lou); qxq@gdut.edu.cn (X. Qiu)

Table of Content

1. Supplementary Methods **1**

1.1. Materials and Analytical Methods 1

1.2. Biomass composition analysis 13

1.3. Lignin isolation from biomass solids and pretreatment liquors 13

2. Supplementary Notes **15**

2.1. Supplementary Note 1: Bond dissociation energy (BDE) calculation 15

2.2. Supplementary Note 2: β–O–4 model compound studies 16

2.3. Supplementary Note 3: Reaction of model compounds bearing C=O with amines in hydrogenolysis 25

2.4. Supplementary Note 4: Reaction of xylose with amines 28

3. Supplementary Figures **29**

4. Supplementary Tables **44**

References **48**

1. Supplementary Methods

1.1. Materials and Analytical Methods

Materials: Diethylamine (≥ 99.0%) was purchased from Damao Co., Ltd. (Tianjin, China). Methanol, acetic acid, and sodium hydroxide were bought from Guangzhou Chemical Reagent Factory. 10% Pd/C, furfural, 5-hydroxymethylfurfural (5-HMF), levulinic acid, xylose, nitrobenzene, and *n*-decane were purchased from Shanghai Aladdin Biochemical Technology Co., Ltd. Guaiacylglycerol-β-guaiacyl ether (GE, ≥ 97%), ammonium hydroxide solution, choline chloride (ChCl), 4-hydroxyacetophenone (98%), 4-hydroxy-3-methoxyphenylpyruvic acid (≥ 95%), and glycerol were purchased from Shanghai Macklin Biochemical Technology Co., Ltd. 4-Hydroxyphenylacetic acid (98%) was purchased from Shanghai Energy Chemical Co., Ltd. 4-Hydroxy-3-methoxycinnamaldehyde (98%) was obtained from Shanghai Bidepharm Co., Ltd. All the chemicals were used without any purification. Commercial Celluclast 1.5L (40 FPU/mL), Cellic® CTec2 (84 FPU/mL), β-glucosidase (β-G) from *Aspergillus niger* (power, ≥ 750 U/g), and sodium polystyrene sulphonates (SPS, standard for GPC) were supplied by Novozymes and Sigma-Aldrich, respectively. The filter paper activity of cellulase was determined according to Ghose *et al.*[1] Enzymatic hydrolysis lignin (EHL) purified from the corncob enzymatic residue was kindly provided by Shandong Longlive Bio-technology Co., Ltd. Corn stover (CS) was kindly provided by COFCO Bio-Energy (Zhaodong) Co., Ltd. and ground to pass through a 20-mesh screen. Corn cob residue (CCR) treated with dilute acid was purchased from Jinan Shengquan Group Share-Holding Co., Ltd. Deep eutectic solvent (DES) was prepared by mixing ChCl and glycerol (1:2, molar ratio), and the mixture was heated at 80 °C with stirring until a clear liquid form.[2]

Fourier transform infrared spectrometer: The FTIR spectra of lignin were recorded on an FTIR

spectrometer from Bruker (TENSOR 27) using the potassium bromide pellet technique.

X-ray diffraction (XRD): All the lignocellulose samples were milled to pass through a 60-mesh screen prior to measurement. The XRD diffraction profile was obtained using a Bruker diffractometer (D8 advance) within the range of 10–40° at a scanning rate of 4°/min. Deconvolution, peak detection, and Gaussian fitting of the measured diffractogram were performed using the PeakFit software. The Bragg angles of 14.8°, 16.3°, 22.4°, and 34.5° were associated with cellulose I. The peak at 20.5° corresponded to amorphous cellulose, with its half-peak width being twice that of the other peaks. The crystallinity index (CrI), indicative of the overall crystallinity in the biomass, was calculated by dividing the area of the crystalline peaks by the total area of the obtained XRD patterns. To minimize the influence of amorphous lignin and hemicelluloses, the CrI-to-cellulose content ratio was employed as an indicator for assessing the crystallinity of cellulose itself.

Elemental analysis: An Elemantar Vario EL cube elemental analyzer was used to determine the C, H, and N contents of lignin.

Contact angle: A contact angle meter (JC2000C1, Powereach, Shanghai, China) was used to measure the water contact angle dissimilarity on different lignin films.

Gel-permeation chromatograph (GPC): The weight-average molecular weight (M_w) and number-average molecular weight (M_n) of lignin were determined using a Waters e2695 system equipped with a 30 cm × 7.8 mm (L. × I.D.) TSKgel GMPWxl column (TOSOH, Tokyo). A variable 2489 UV/Vis detector at wavelengths of 280 nm and 254 nm was used for the detection. Utilizing a 0.1 M NaOH aqueous solution

as the mobile phase allowed direct detection of the liquids from DEA and NaOH pretreatment before and after hydrogenolysis, without the need of additional post-treatments such as acetylation. The flow rate of eluent was maintained at 1.0 mL/min, and the column temperature was set to 30 °C. For establishing the calibration curve, sodium polystyrene sulphonate (SPS) standards (210–17000 Da) and guaiacylglycerol-β-guaiacyl ether were used as standards. A 3rd order fit type was employed to relate apparent molecular weight to retention time.

Quartz crystal microbalance with dissipation monitoring (QCM-D): Lignin films were prepared on gold-coated QCM sensors. Prior to film preparation, the QCM gold sensors were immersed in a solution of 25% ammonia, 30% hydrogen peroxide, and water (1:1:5, v/v/v) while being treated with an ultrasonicator. They were then rinsed with deionized water and dried under a flow of nitrogen gas. After the cleaning process, 2 wt% lignin solutions were prepared by fully dissolving lignin samples in ammonia solution. These prepared lignin solutions were then added onto the crystal sensors, and thin lignin films were coated onto the QCM sensors using a spin coater (WS-400Bz-6NPP-LITE, Mycro Technologies Corp., China).

The binding of cellulase (Celluclast 1.5L) to the lignin films was monitored *in situ* using a QCM-D (Q-Sense E1 instrument, Q-Sense AB, Sweden). The experiments were performed at 40 °C in a pH 4.8 acetate buffer (50 mM). Upon contact with the buffer solution, the film underwent swelling until equilibrium was reached. Subsequently, the enzyme solution (0.1 g/L) was continuously injected at a flow rate of 0.15 mL/min. Due to its robust stability and repeatability, the frequency shifts in the third overtone (Δf_3) were commonly used to reflect the adsorption ability. The cellulase adsorption capacity on the lignin film is calculated as follows:

$$\Delta m = -\frac{C\Delta f_3}{3} \tag{1}$$

Where Δm means the adsorption mass change, ng·cm^{-2}; C denotes the mass sensitivity constant with a value of ng·cm^{-2}·Hz^{-1}; Δf_3 denotes the change in the third overtone for frequency, Hz.

Scanning Electron Microscopy (SEM): The morphology of CS was characterized using a scanning electron microscope (Hitachi SU8220, Japan) after sputtering with a layer of gold.

X-ray photoelectron spectroscopy (XPS): The N content in lignin power was quantified using an X-ray photoelectron spectrometer (AXIS SUPRA+, SHIMADZU).

High-Performance Liquid Chromatography (HPLC): An HPLC (Agilent 1260 Infinity II) equipped with an HPX-87H column was used to determine the monosaccharide concentration. The program setup was as follows: 5 mM H_2SO_4 as an HPLC mobile phase, a flow rate of 0.5 mL/min, a column oven temperature of 50 °C, and a detector temperature of 50 °C.

HPLC-MS: Analysis was performed on an Agilent1290 / Bruker maXis impact. The program for HPLC is shown below.

The gradient elution program in HPLC-MS.

Time (min)	Methanol (v%)	0.1% Formic acid (v%)
0	30	70
10	30	70
40	95	5

50	95	5
55	5	95
60	5	95

Two-dimensional heteronuclear-single-quantum-coherence spectra (2D HSQC NMR): Lignin solutions were prepared by dissolving 100 mg of lignin power in 600 μL of DMSO-d_6. The 2D HSQC spectra of the lignin were recorded on a Bruker AVANCE III HD 600 MHz spectrometer with a scanning time of 8 h. The semi-quantitative assessment of lignin inter-linkages was conducted based on a cluster of signals representative of all C9 units.

$$I_{C9}\ \text{units} = 0.5I_{S2,6} + I_{G2} + 0.5I_{H2,6} \quad (2)$$

Where I_{C9} denotes the integral value of the aromatic ring; $I_{S2,6}$, I_{G2}, and $I_{H2,6}$ represent the integral value of S, G, and H units, respectively. According to the internal standard (I_{C9}), the amount of linkages could be calculated by the following formula:

$$X = I_x/I_{C9} \quad (3)$$

Where X denotes the linkage amount per 100 C9 units; I_X is the integral value of the α-position in A (β–O–4′), B (β–5′), and C (β–β′) subunits.

Gas chromatography-mass spectrometric (GC-MS): The prepared sample was analyzed using GC-MS with an Agilent 5975C-7890A series GC equipped with an HP-5ms capillary column (30 m × 250 μm, L. × I.D., 0.25 μm film thickness). The injection port was set at 300 °C, and helium was used as the carrier gas at a flow rate of 1 mL/min with a split ratio of 20:1. The following temperature-rising program was used: the

GC oven temperature profile initially held at 50 °C for 2 min, then increased to 180 °C at a rate of 8 °C/min, followed by a ramp of 10 °C/min to 300 °C, where it was held for 5 min.

Gas chromatography with flame-ionization detection (GC-FID): The prepared sample was analyzed using a GC (SHIMADZU GC-2018 series) equipped with an HP-5MS column and a flame ionization detector (FID). The injection temperature was set to 300 °C, and nitrogen was used as the carrier gas at a flow rate of 30 mL/min. The column temperature program was as follows: an initial isothermal temperature of 50 °C for 2 min, followed by a ramp at a rate of 8 °C/min up to 180 °C, then a ramp at a rate of 10 °C/min to 230 °C (maintained for 5 min), and finally a ramp at a rate of 10 °C/min to 280 °C, where it was held for 5 min. The yield of the identified phenolic monomer was determined based on an internal standard (*n*-decane) and the effective carbon number (ECN) method. The weight fraction of each compound in the sample was calculated using the following formula:

$$n_{\text{decane}} = \frac{W_{\text{decane in sample}}}{MW_{\text{decane}}} = \frac{0.5\ \text{mg}}{142\ \text{mg/mmol}} = 0.0035\ \text{mmol} \quad (4)$$

$$n_{\text{monomer}} = \frac{A_{\text{monomer in sample}}}{A_{\text{decane in sample}}} \times 0.0035\ \text{mmol} \times \frac{ECN_{\text{decane}}}{ECN_{\text{monomer}}} \quad (5)$$

$$Y_{\text{phenolic monomer}} = \frac{n_{\text{monomer}} \times MW_{\text{monomer}}}{W_{\text{lignin in pretreatment liquor}}} \times 100\% \quad (6)$$

In the equations,

$W_{\text{decane in sample}}$ (mg): the weight of decane added to each analyzed sample;

MW_{decane} ($\text{mg}\cdot\text{mmol}^{-1}$): the molecular weight of decane (142 $\text{mg}\cdot\text{mmol}^{-1}$);

n_{decane} (mmol): the molar amount of decane added in the sample;

n_{monomer} (mmol): the molar amount of phenolic monomer in the sample;

$A_{\text{monomer in sample}}$: the peak area of monomer in the GC-FID chromatogram;

$A_{\text{decane in sample}}$: the peak area of decane in the GC-FID chromatogram;

ECN_{decane}: the effective carbon number (10) of decane;

$ECN_{monomer}$: the effective carbon number of the phenolic monomer;

$Y_{phenolic\ monomer}$ (wt%): the weight yield of phenolic monomer;

$MW_{monomer}$ (mg·mmol^{-1}): the molecular weight of monomer;

$W_{lignin\ in\ pretreatment\ liquor}$ (mg): the weight of lignin in 20 mL DEA and NaOH pretreatment liquor are 68 mg and 83 mg, respectively. 20 mL is the volume of pretreatment liquor used in hydrogenolysis.

Comprehensive two-dimensional gas chromatography time-of-flight mass spectrometry (GC × GC-TOF-MS): The sample was analyzed using an Agilent 8890 GC coupled with an Agilent 7250A TOF. The GC × GC is equipped with a DB-5MS column (60 m × 0.25 mm × 0.25 μm) as the first dimension column connected to a DB-17MS (1.0 m × 0.25 mm × 0.15 μm) as the second dimension column through an SV (C7-C40) connection. The GC system operated under programmed temperature conditions: starting at 40 °C for 5 min, ramping up to 310 °C at a heating rate of 3 °C/min, and holding at 310 °C for 20 min. The injection port was set at 300 °C, and helium served as the carrier gas at a constant flow rate of 1.0 mL/min without splitting. The modulation period was 7 s. Data acquisition was performed at a rate of 50 spectra per second within a scanning range of 30 to 650 amu. The GC-TOF-MS interface (transfer line) temperature was maintained at 280 °C, while the ion source temperature was set at 230 °C. TOF-MS detection was carried out in HES mode (−70 eV). The yield of N-containing aromatic monomer identified by GC × GC-TOF-MS was calculated using the published method for quantifying aromatic products.[3] For pyridine derivatives, the GC-FID chromatogram only displayed peaks for 2-methyl-5-ethylpyridine and 3-ethyl-4-methylpyridine due to limited resolution. As a result, quantification of pyridine derivatives was accomplished through the following approach: first, using GC-FID, the weight of 2-methyl-5-ethylpyridine

and 3-ethyl-4-methylpyridine in the sample was determined based on an internal standard (*n*-decane) and the ECN method. Subsequently, the relative response factor (*RRF*) for the pyridine derivative in the GC × GC-TOF-MS contour chromatogram was calculated using the following equation:

$$RRF = \left(\frac{m_1 \times A_{IS}}{m_{IS} \times A_1} + \frac{m_2 \times A_{IS}}{m_{IS} \times A_2}\right)/2 \quad (7)$$

Where m_1 and m_2 are the mass of 2-methyl-5-ethylpyridine and 3-ethyl-4-methylpyridine in the sample, respectively; A_1 and A_2 are the peak area of 2-methyl-5-ethylpyridine and 3-ethyl-4-methylpyridine in the GC × GC-TOF-MS contour chromatogram; m_{IS} denotes the mass of *n*-decane added, and A_{IS} is the peak area of *n*-decane in the GC × GC-TOF-MS contour chromatogram.

In the end, the weight fraction of each identified pyridine derivative was calculated using the following formula:

$$Y_{\text{pyridine derivative}} = \left(RRF \times \frac{A_i}{A_{IS}} \times m_{IS}\right)/W_{\text{lignin in pretreatment liquor}} \quad (8)$$

Where $Y_{\text{pyridine derivative}}$ is the weight fraction of pyridine derivative **i**; A_i is the peak area of pyridine derivative **i** in the GC × GC-TOF-MS contour chromatogram.

Techno-economic analysis (TEA) methodology: Techno-economic models include a conceptual level of process design to develop a detailed process flow diagram, rigorous materials and energy balance calculation, capital and project cost estimation, a discounted cash flow economic model, and the calculation of an MPSP.[4, 5, 6]

Nitrobenzene oxidation method: Nitrobenzene oxidation of DEA-L and AL were conducted following previously established procedures.[7] 40 mg of dried lignin was dissolved in a mixture of 2 N NaOH (4 mL) and nitrobenzene (0.24 mL) in a Teflon-lined reactor and heated in an oven at 170 °C for 1 h. The reaction

mixture was centrifuged and then extracted with ethyl acetate (30 mL × 3) to remove unreacted nitrobenzene. The alkaline water layer was acidified to pH 2 with a 2 N HCl solution and extracted with ethyl acetate (30 mL × 3). The organic layer was washed with brine and dried over anhydrous Na_2SO_4, and the solvent was removed by rotary evaporation. The resultant products were dissolved in 2 mL of acetone with decane as an IS. Silylations of the sample solution were performed with BSTFA prior to GC-MS analyses.

$$Theoretical\ maximum\ yield\ (\%) = \frac{m_{\text{monophenols}}\ (\text{mg})}{40\ \text{mg} \times \text{lignin purity}\ (\%)} \tag{9}$$

Mass balance of DEA: The DEA consumed in residual and dissolved lignin was calculated based on the N content of DEA-treated corn stover (N: 0.23%) and DEA-L (N: 3%), respectively. The loss of 40% DEA was assessed by recording the weight changes in the overall material before and after pretreatments. To figure out DEA loss in hydrogenolysis, HPLC equipped with an RID was employed to analyze the DEA content variations in a 4% DEA solution under hydrogenolysis conditions. For the determination of DEA involved in the amination of hydrogenolysis products (including all aminated compounds), the dried hydrogenolysis products obtained through rotary evaporation and subsequent freeze-drying were subjected to an N elemental analysis (N: 5.2%).

Molecular electrostatic potential: The computations were performed using Materials Studio 2017 software and its DMol3 module. All molecular geometries were optimized using the B3LYP level of theory with a maximum of 50 iterations. After analysis using DMol3, molecular electrostatic potential diagrams of DEA molecules in various solution environments were generated.

Molecular dynamics simulations: Materials Studio 2017 software and its Forcite module were used for the

computations. The lignin model used consists of nine phenylpropanoid structural units and five types of representative interunit linkages, including β–O–4′, α–O–4′, β–β′, β–5′, and 5–O–4′.[8] The optimized geometry of the lignin structure using B3LYP/6-31 G (d, p) level of theory in Gaussian 09 software is shown below. To study non-bonded interactions between lignin and DEA in a 40% v/v DEA solution, the system containing an amorphous cell with one lignin molecule, one hundred DEA molecules, and eight hundred water molecules was constructed. Periodic boundary conditions were applied, using the COMPASS force field and Ewald method to control electrostatic interactions. Geometry optimization for the constructed system was carried out with a maximum of 10,000 iterations. Then, the NVT ensemble with a period of 400 ps was used to further equilibrate the system. Finally, simulations ran for 2 ns under the NPT system at a temperature of 403 K. The last 500 ps of the simulation were extracted for analysis of the radial distribution function (RDF).

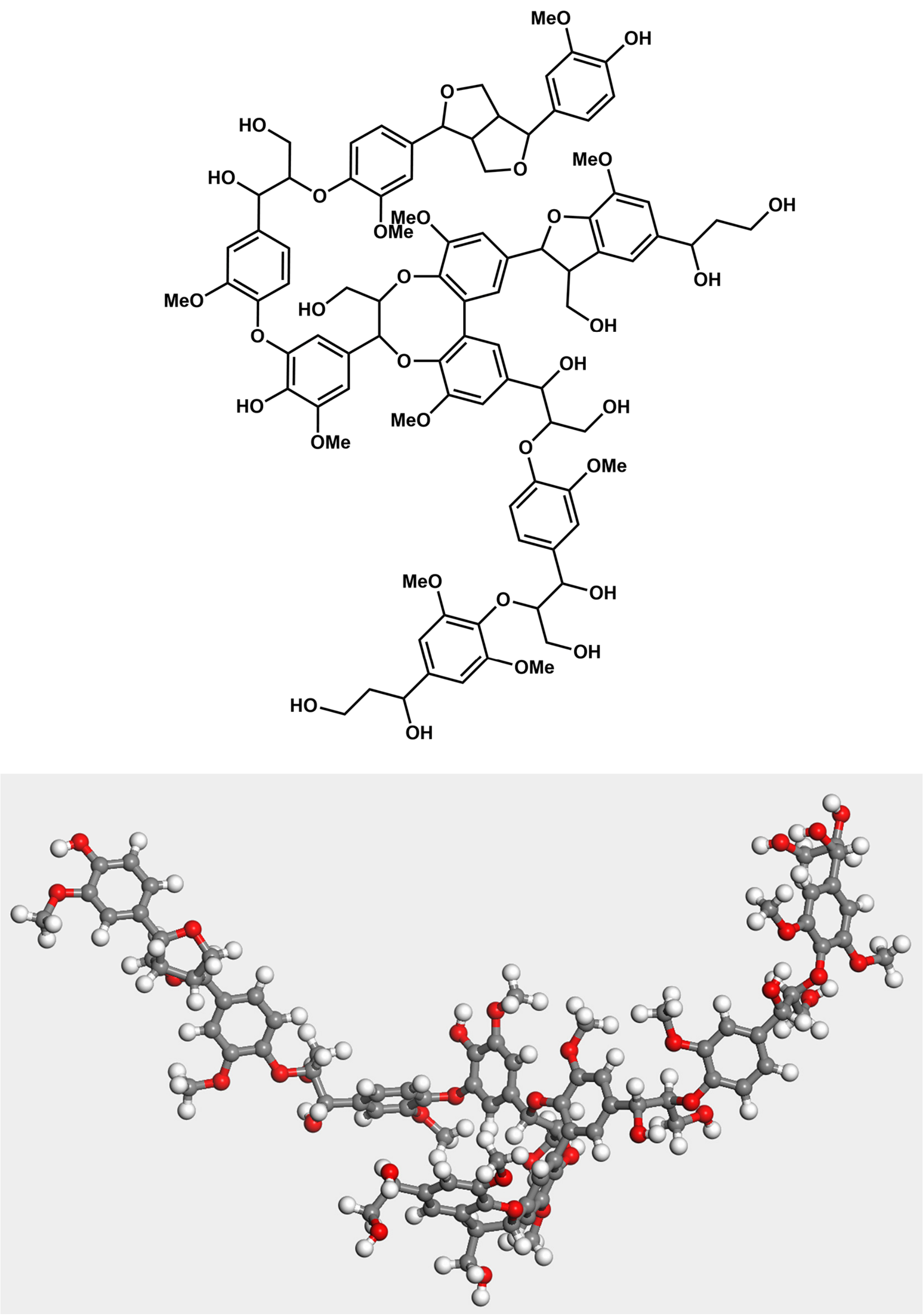

The optimized structure of the lignin model.

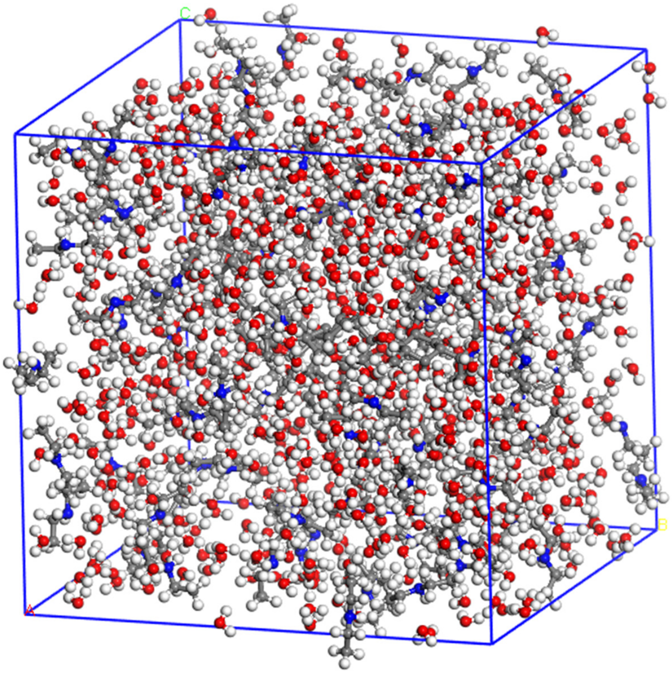

Lignin: 1

DEA: 100

H_2O: 800

The system models of lignin in DEA aqueous solution.

1.2. Biomass composition analysis

Determination of ash in biomass: About 0.6 g of biomass solid was placed into a muffle furnace. The muffle furnace was operated under programmed temperature conditions: ramp from room temperature to 105 °C and kept for 10 min, then ramp to 250 °C and hold for 30 min, and finally ramped to 575 °C and hold for 8 h. As it cooled, the ash remaining was collected and weighed. The ash content in raw CS was 9.0%.

Determination of structural carbohydrates and lignin in biomass: Structural carbohydrate and lignin content in biomass were analyzed by two-step acid hydrolysis according to the standard laboratory analytical procedures (LAPs) of the National Renewable Energy Laboratory (NREL). In detail, 0.3 g of biomass solid was mixed with 3.0 mL 72wt% H_2SO_4 in the pressure bottle (100 mL), and the mixture was shaken at 200 rpm and 30 °C for 60 min. After that, the acid was diluted to a 4% concentration by adding 84.0 mL of deionized water, and the acid hydrolysis was performed in an autoclave at 121 °C for 60 min. In order to correct sugar losses, 10 mL of sugar recovery standard (SRS) containing 1.2 g/L glucose and 0.6 g/L xylose was mixed with 348 μL of 72 wt% H_2SO_4 and placed into the autoclave together with the test samples. After the reaction finished, 1 mL of the supernatant was taken for sugar and acid-soluble lignin concentration determination by an HPLC and an UV-Visible spectrophotometer (320 nm), respectively. Acid insoluble residue was dried at 105 °C for 4 h at least, and the weight was recorded. The raw CS is composed of 39.7% cellulose, 22.5% hemicelluloses, 13.8% acid-insoluble lignin (AIL), and 1.8% acid-soluble lignin (ASL). Here, lignin content is the result after deducting ash content. The cellulose and AIL content of corn cob residue (CCR) are 70.8% and 25.9%, respectively.

1.3. Lignin isolation from biomass solids and pretreatment liquors

Lignin isolation from biomass solids: The isolation of cellulolytic enzyme lignin (CEL) from untreated CS

and the residual lignin from DEA-treated corn stover (DEA-RL) was conducted according to the published procedure.[9] Briefly, the lignocellulosic solids were ground in a ball mill at 400 rpm for 4 h in total and in the intervals of 30 min working and 10 min pause. Subsequently, the ball-milled solid was enzymatically hydrolyzed with a Cellic® CTec2 dosage of 0.3 mL/g solid and 10% w/v biomass loadings for 24 h at 50 °C, pH 4.8, and 150 rpm. The hydrolyzed residues were then mixed with dioxane/water (9:1, v/v) at 35 °C for 48 h to extract lignin. After removing the solvent from the organic phase with a rotary evaporator, the rude lignin was redissolved in acetic acid/water (9/1, v/v), and then re-precipitated in plenty of water. Finally, the precipitations were water-washed, and subjected to freeze-drying to obtain target lignin samples.

Lignin isolation from pretreatment liquors: DEA-treated lignin (DEA-L) from the corresponding pretreatment liquor was isolated using the following procedure: The liquor was acidified with hydrochloric acid (10%) to pH = 2 and then centrifuged to collect the precipitate. After being washed with deionized water, the precipitate was freeze-dried to give DEA-L.

Alkaline lignin (AL) from the NaOH pretreatment liquor was isolated with the same procedure above. In brief, 5.4 g of CS was treated with 4% w/w NaOH at a biomass loading of 10% w/w and 130 °C for 1 h. After the reaction, the solid substrate was separated from the NaOH pretreatment liquor and then washed twice with 10 mL of water per gram of solid. The NaOH pretreatment liquor was combined with the washings. The mixture was then acidified to isolate AL.

2. Supplementary Notes

2.1. Supplementary Note 1: Bond dissociation energy (BDE) calculation

The geometry optimization of the lignin dimeric model was performed using the 6-311++G(d,p) basis set in the Gaussian 16 software. The density functional theoretical (DFT) approach was employed with the M06-2X hybrid exchange-correlation functional. BDEs were predicted as the difference in the enthalpy of the molecule and the two dissociated fragments.[10]

BDE calculation for the β–O–4′ motif before and after amination.

<table>
<tr><th>Substance</th><th>Bond dissociation energy</th></tr>
<tr><td>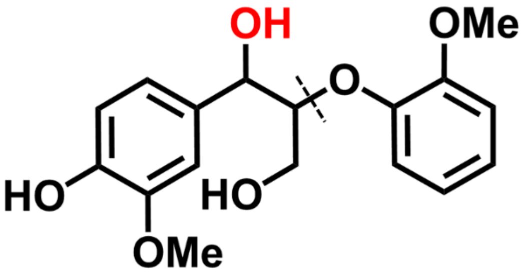
</td><td>307 KJ/mol</td></tr>
<tr><td>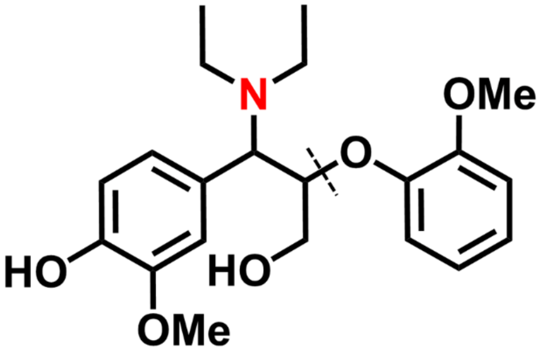
</td><td>297 KJ/mol</td></tr>
</table>

2.2. Supplementary Note 2: β–O–4 model compound studies

In a 50-mL reactor, 25 mg of guaiacylglycerol-β-guaiacyl ether (GE) was mixed with 4 mL of a 40% v/v DEA aqueous solution. The reaction was carried out in an oven at 130 °C for 1 h, followed by drying with a rotary evaporator. 0.8 mL of methanol was added to dissolve the dried residue, and then 120 μL of the solution was sampled for silylation. In detail, the sampled solution was mixed with 500 μL of N-methyl-N-trimethyl-silyl-trifluoroacetamide (MSTFA), 400 μL of pyridine, and 220 μL of the internal standard solution (10 mg decane dissolved in 10 mL methanol) and kept at 40 °C for 80 min. After the reaction, the sample was analyzed using GC-MS. However, due to the complexity of products and the presence of dimers, the NIST library was unable to identify some products. Therefore, these compounds were assigned based on a detailed analysis of their mass fragmentation patterns, as shown below.

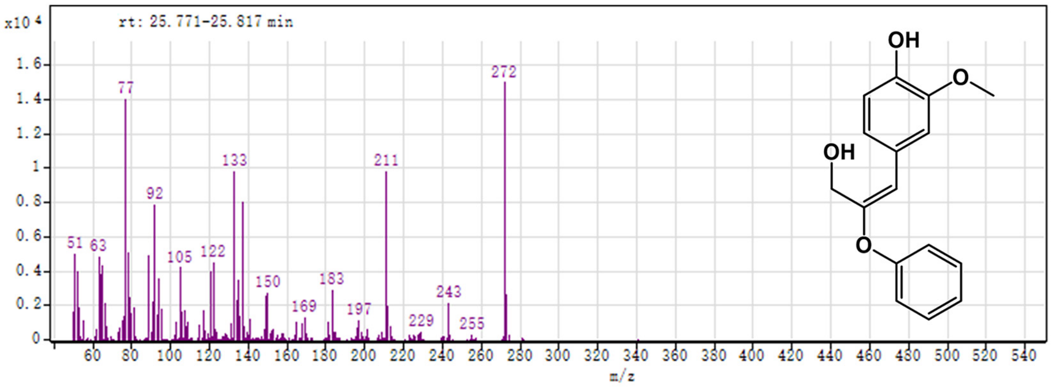

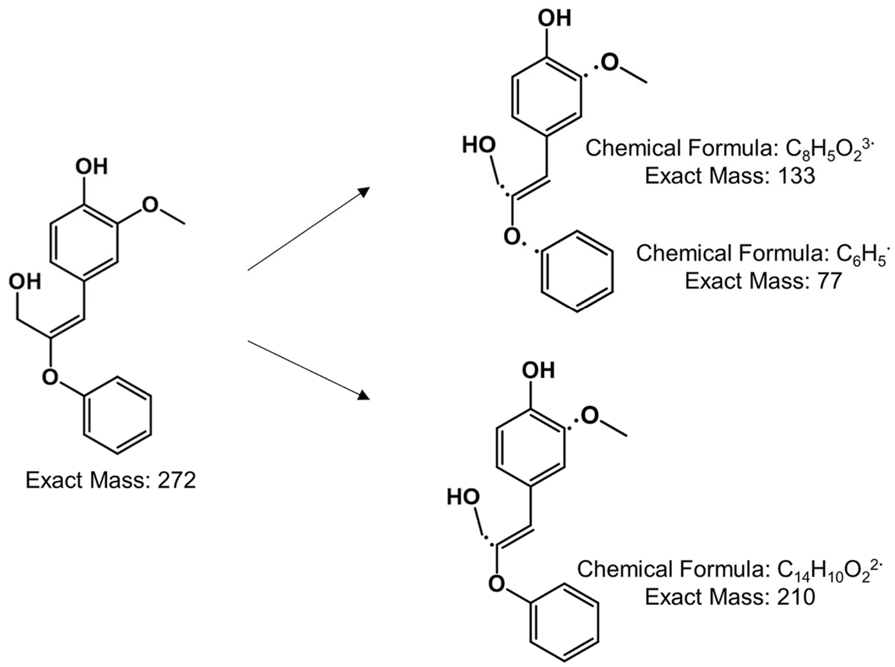

Mass spectrum and fragmentation pattern analysis of dimer **D3.**

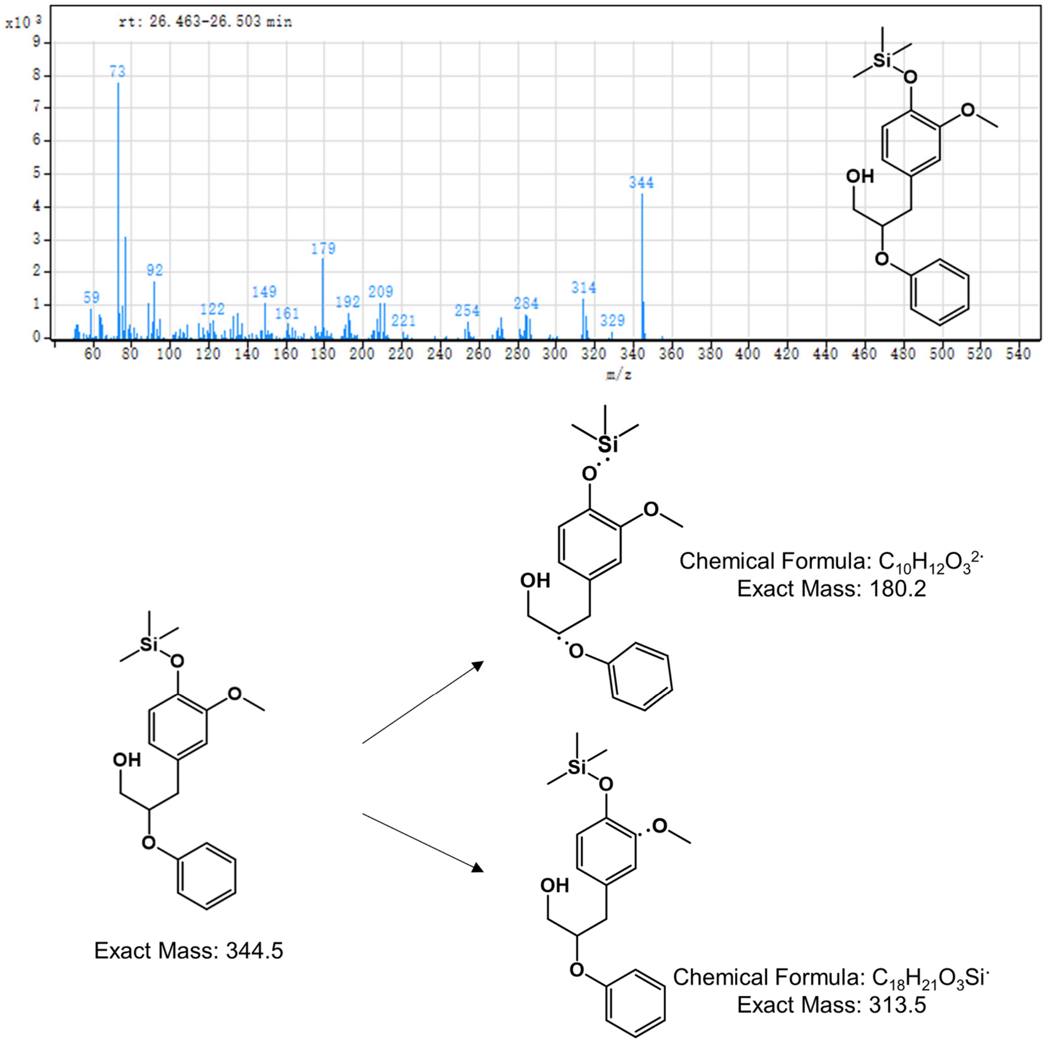

Mass spectrum and fragmentation pattern analysis of dimer **D4.**

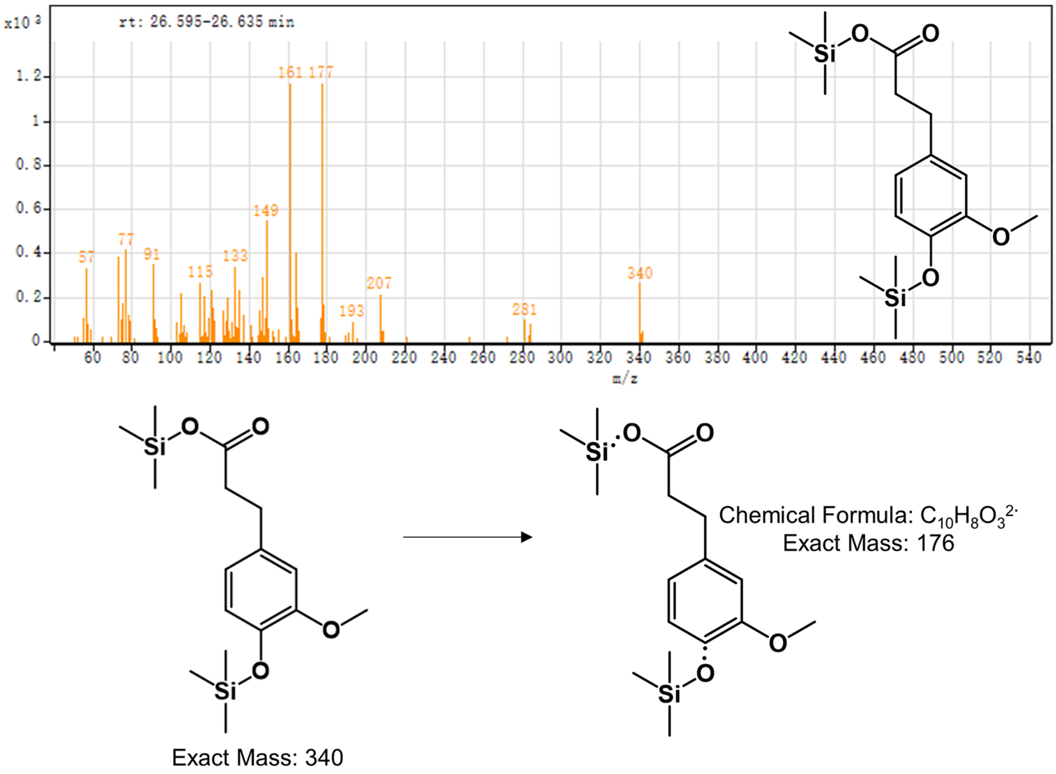

Mass spectrum and fragmentation pattern analysis of monomer **D5.**

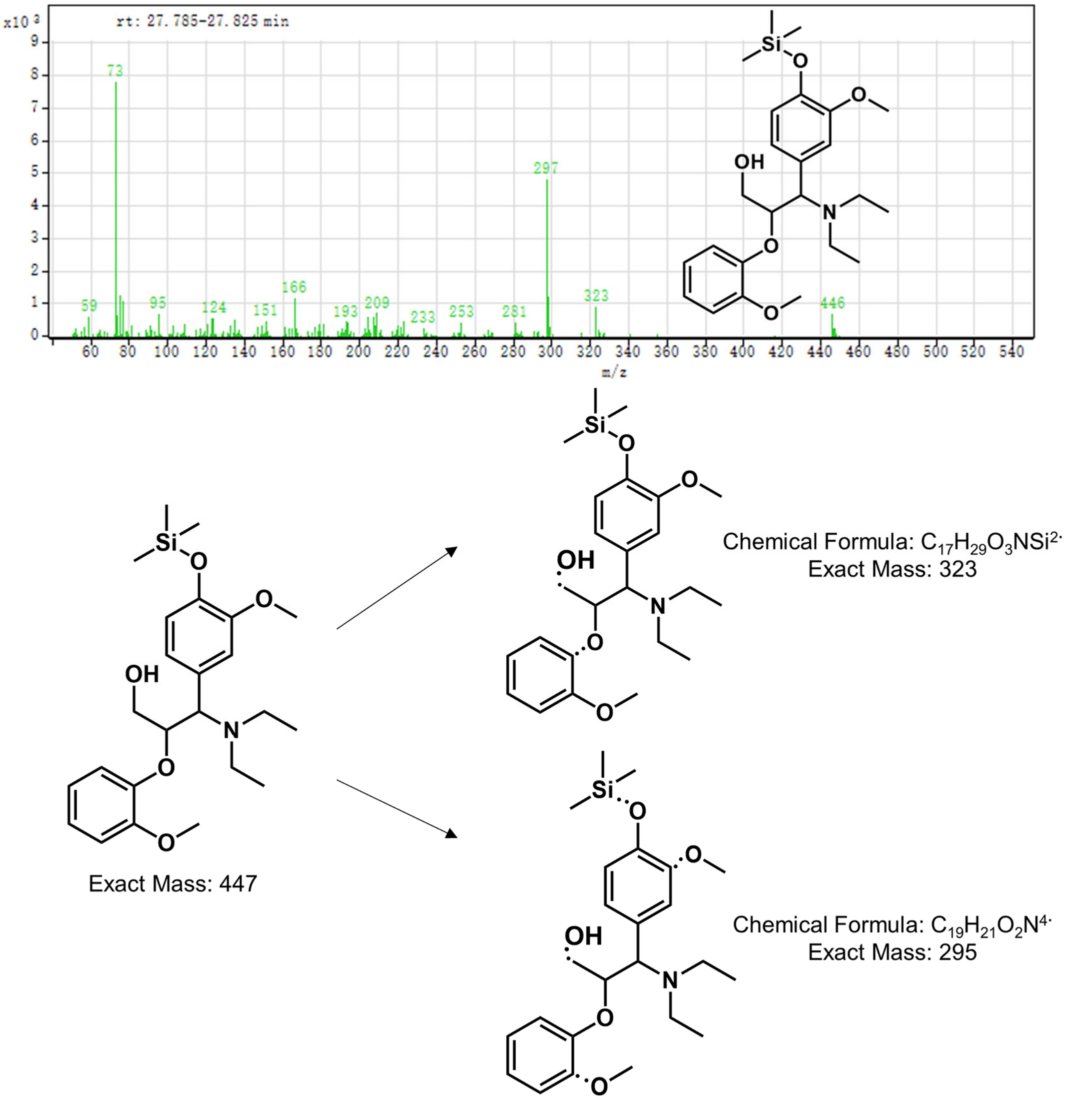

Mass spectrum and fragmentation pattern analysis of dimer **D8.**

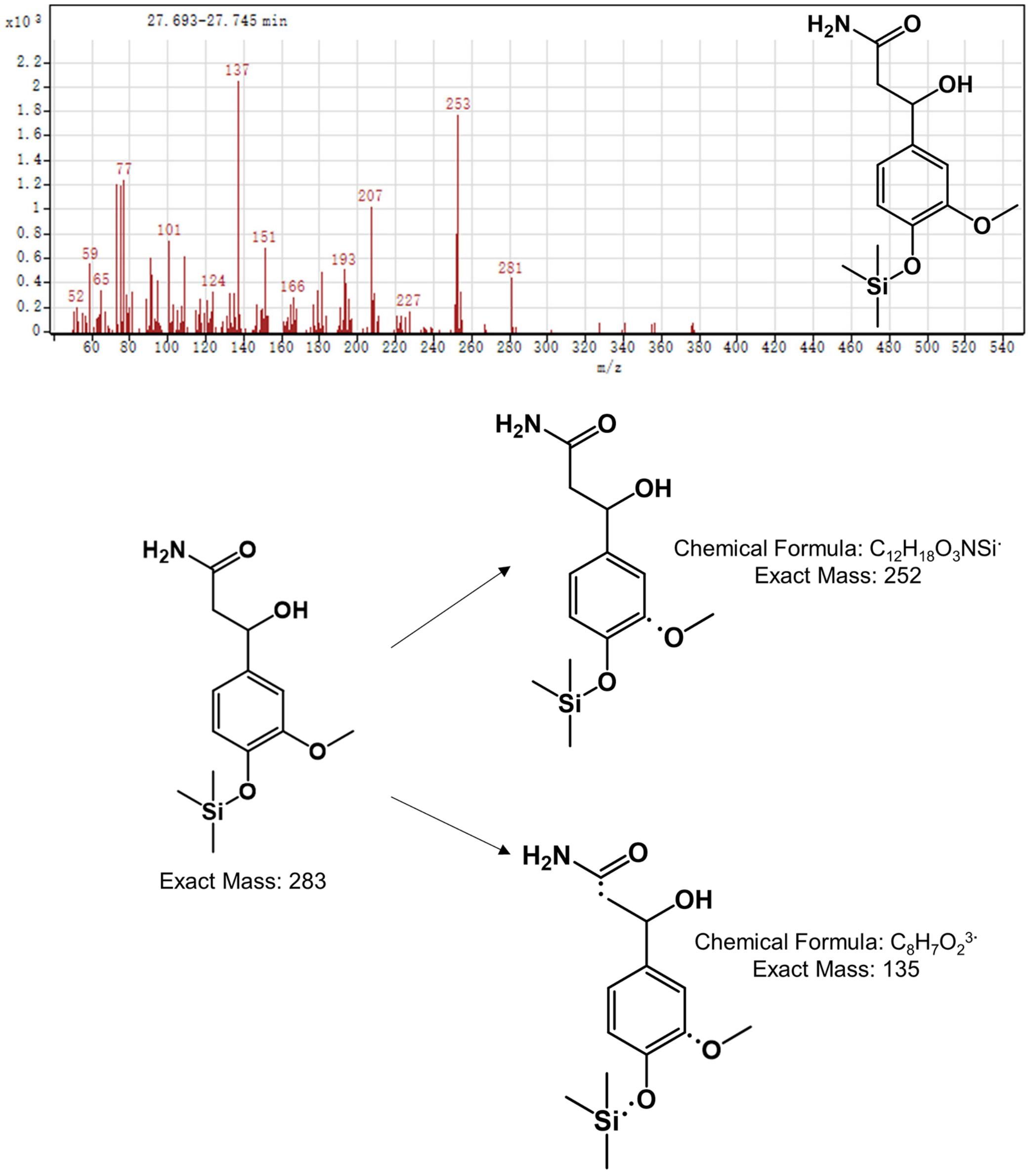

Mass spectrum and fragmentation pattern analysis of monomer **D7.**

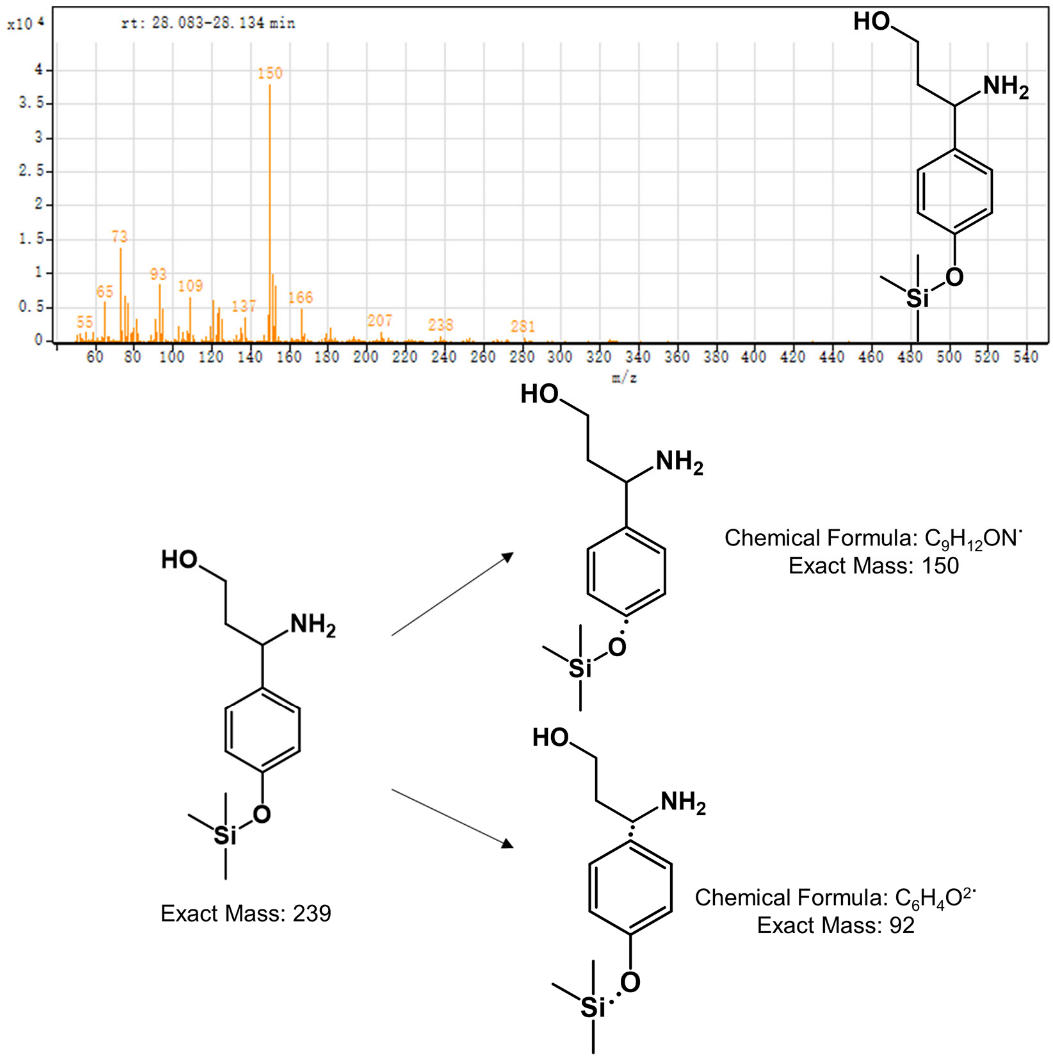

Mass spectrum and fragmentation pattern analysis of monomer **D9.**

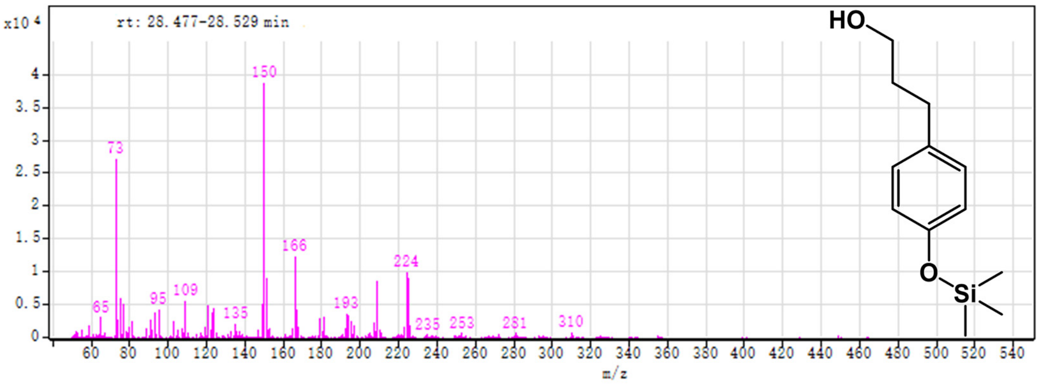

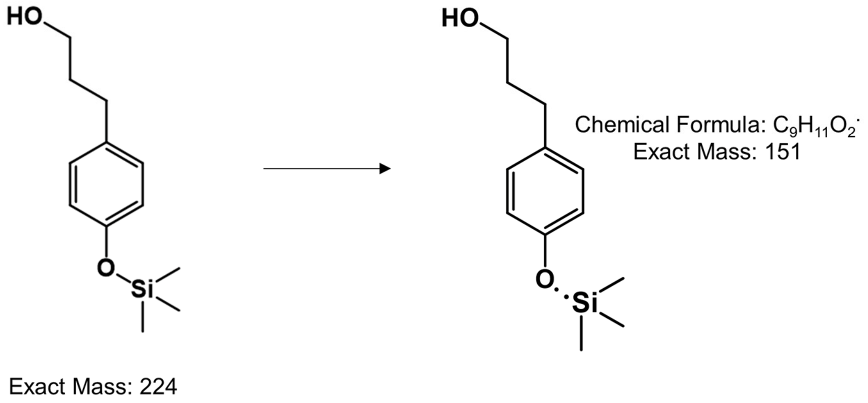

Mass spectrum and fragmentation pattern analysis of monomer **D10.**

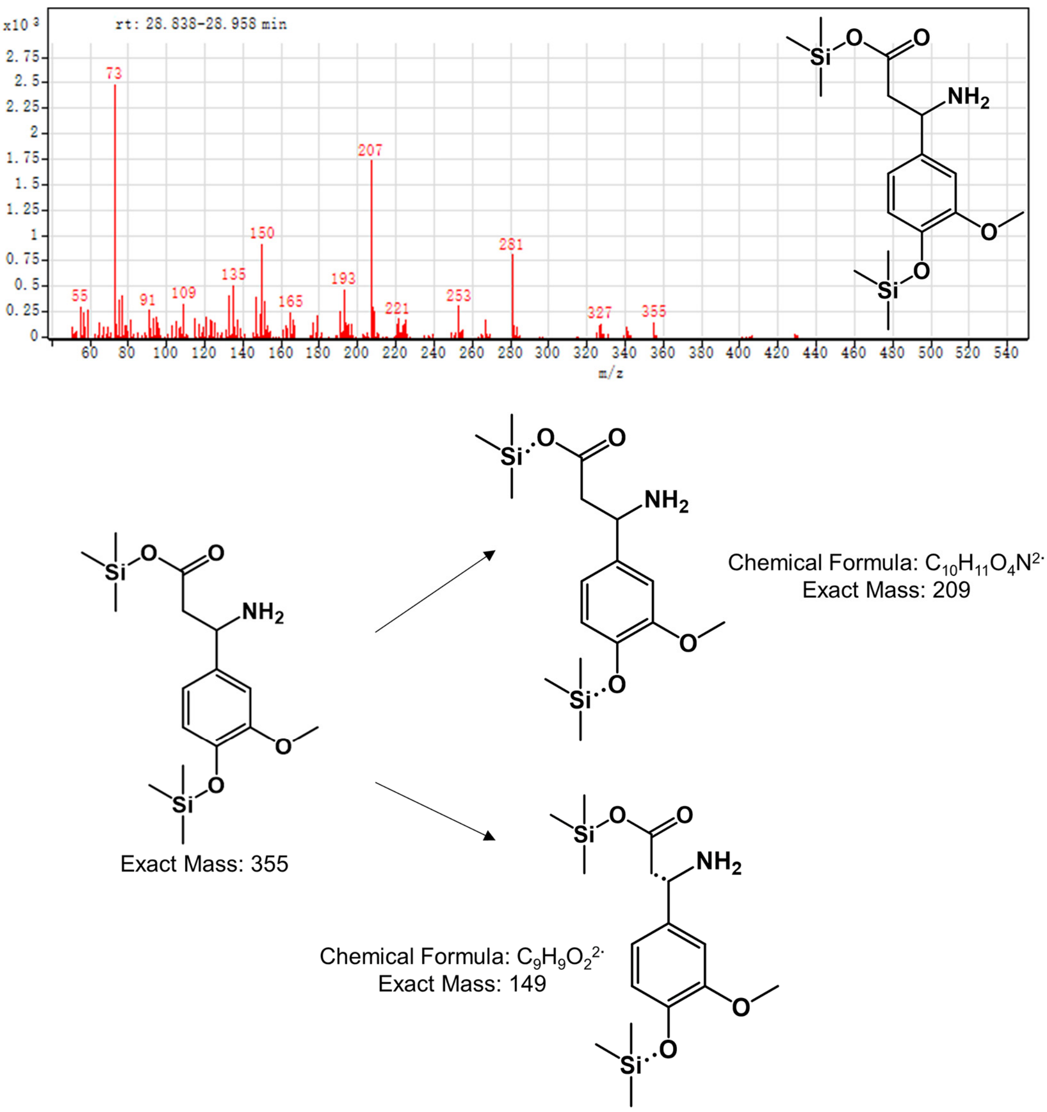

Mass spectrum and fragmentation pattern analysis of monomer **D11.**

2.3. Supplementary Note 3: Reaction of model compounds bearing C=O with amines in hydrogenolysis

In order to prove the hypothesis that pyridine bases were produced from the reaction between the side chains of aldehyde ketones in lignin and amine. Four model compounds bearing C=O groups, 4-hydroxyacetophenone (15 mg), 4-hydroxy-3-methoxyphenylpyruvic acid (15 mg), 4-hydroxyphenylacetic acid (15 mg), and 4-hydroxy-3-methoxycinnamaldehyde (15 mg) were chosen and mixed with 40 mg of 10% Pd/C and 60 mg of copper acetate monohydrate in 20 mL of 5% v/v DEA. The hydrogenolysis conditions were as follows: 10 bar H_2, 250 °C, 60 min, and 400 rpm. After hydrogenolysis, the resultant mixture was acidified to pH 2 with 10% HCl. Subsequently, ethyl acetate (40 mL × 3) was added to extract phenolic monomers into the organic phase and leave N-containing monomers in the aqueous phase. The organic and aqueous phases were dried with rotary evaporation. 3 mL of acetone and 0.5 mL of *n*-decane (1.0 mg/mL in methanol, internal standard) were added to dissolve the dried products from the organic phase, while the dried products from the aqueous phase were dissolved in 2 mL of acetone and 0.4 mL of internal standard. Then, magnesium sulfate anhydrous was added for dehydration. Finally, each sample was injected for GC-MS analysis after passing through a 0.22 μm membrane filter. The GC-MS results of products from the organic and aqueous phases are summarized below.

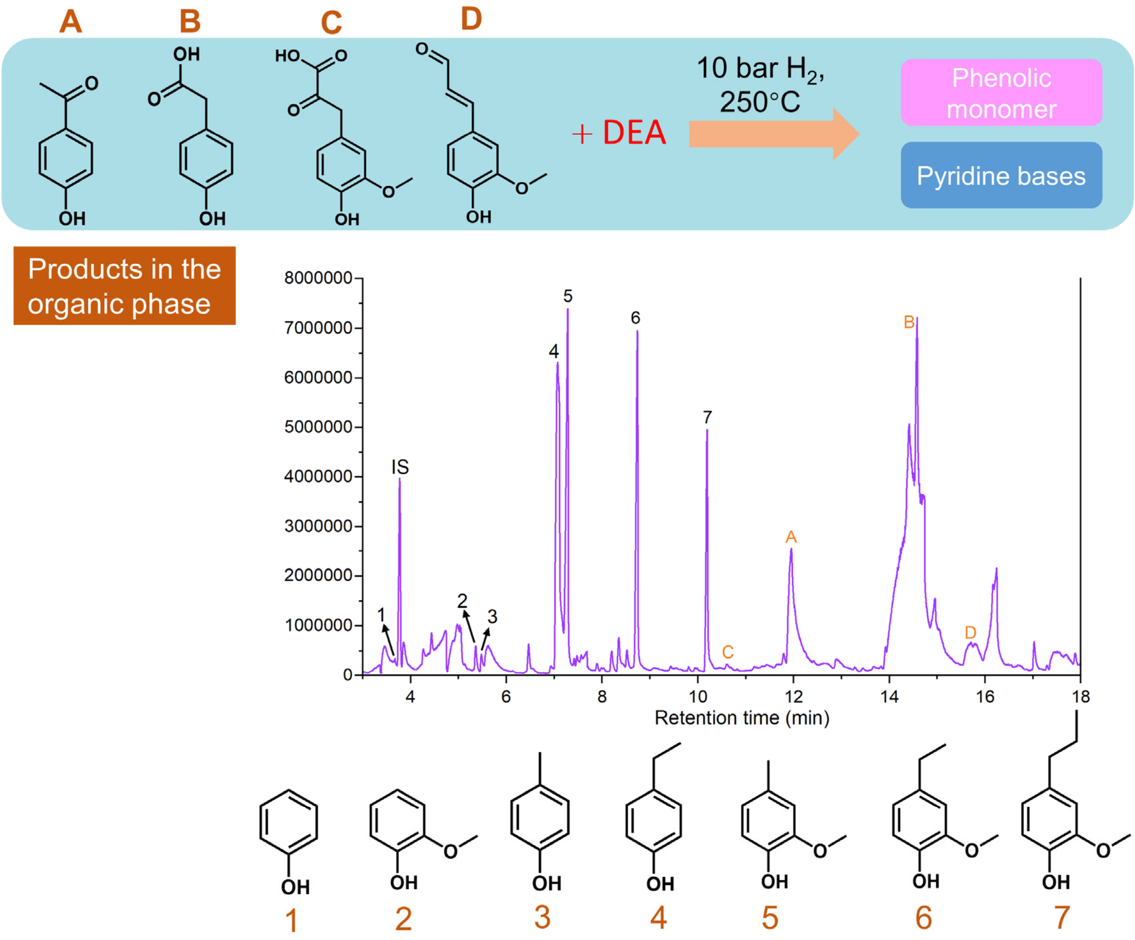

Phenolic products from model reactions.

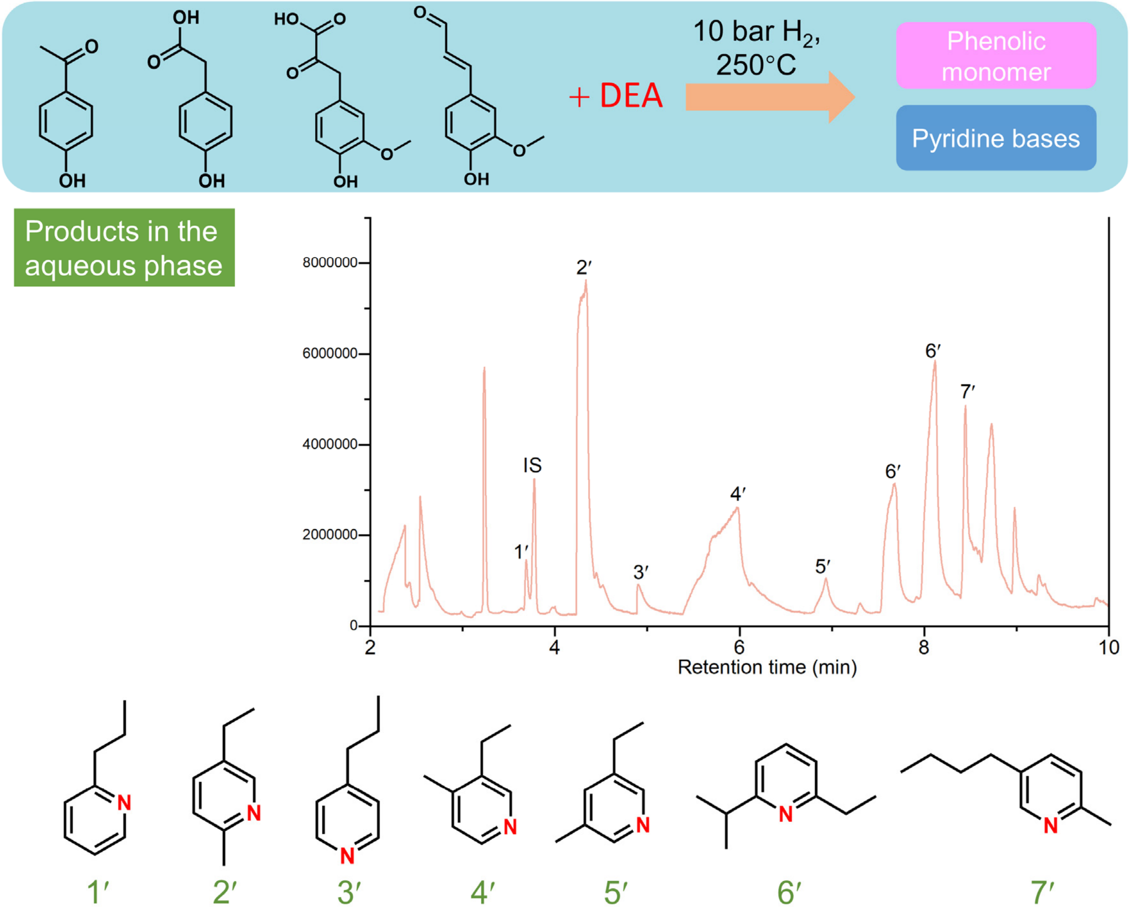

Pyridine base products from model reactions.

2.4. Supplementary Note 4: Reaction of xylose with amines

5 g of xylose was weighed and mixed with DEA (40% v/v) at a solid loading of 30 wt%. The reaction was performed at 130 °C for 1 h. After completion, 30 mL of water was added, and the mixture was extracted with ethyl acetate. The resultant organic phase was dried using a rotary evaporator. Following this, the dried products were dissolved in acetone, which included an internal standard, and subjected to analysis using GC-MS.

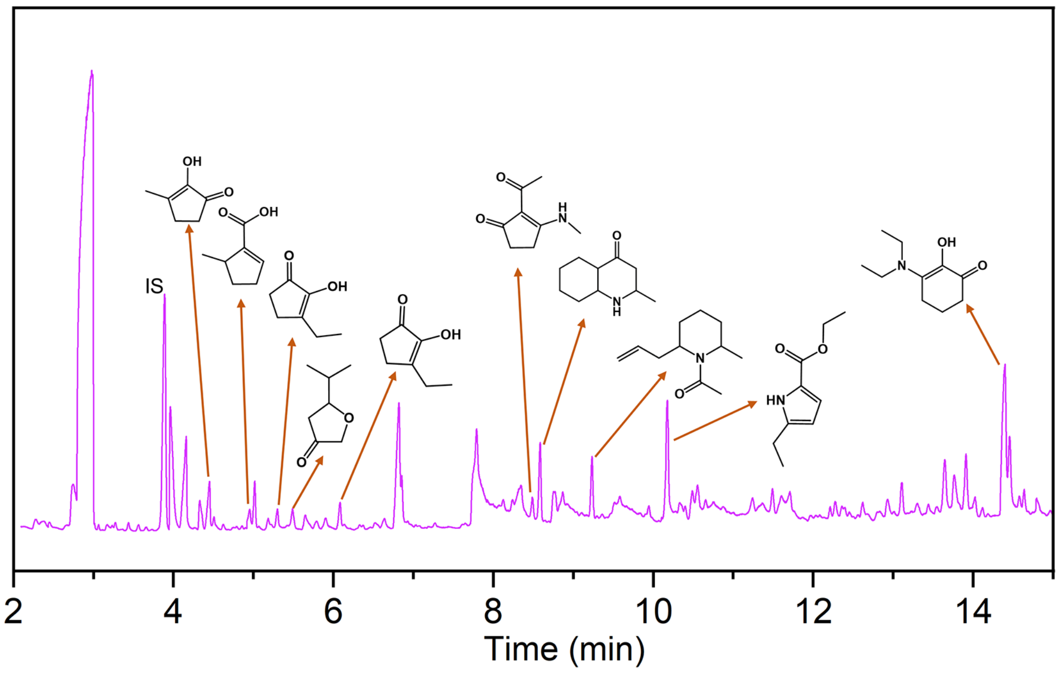

The GC-MS result of the products from the reaction of xylose in 40% v/v DEA.

3. Supplementary Figures

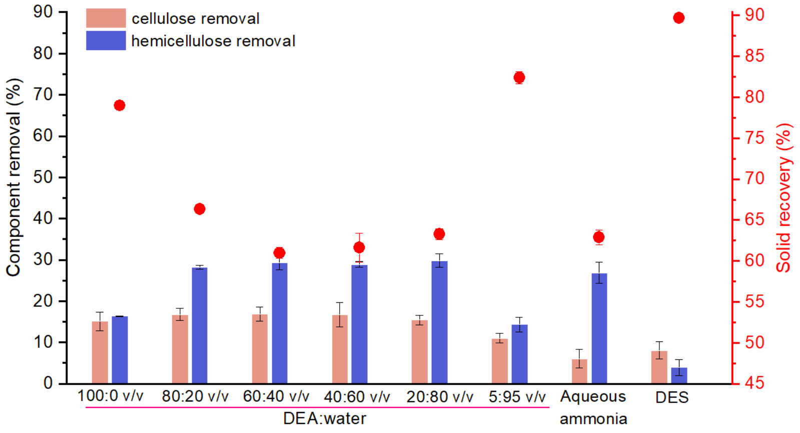

Supplementary Figure 1. Carbohydrate removal and solid recovery in different pretreatments. Error bars represent the standard deviation.

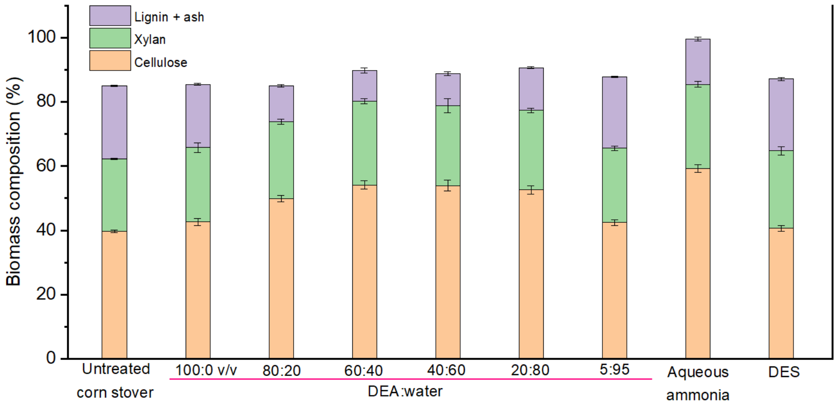

Supplementary Figure 2. Composition analysis of the untreated and pretreated corn stover. Error bars represent the standard deviation.

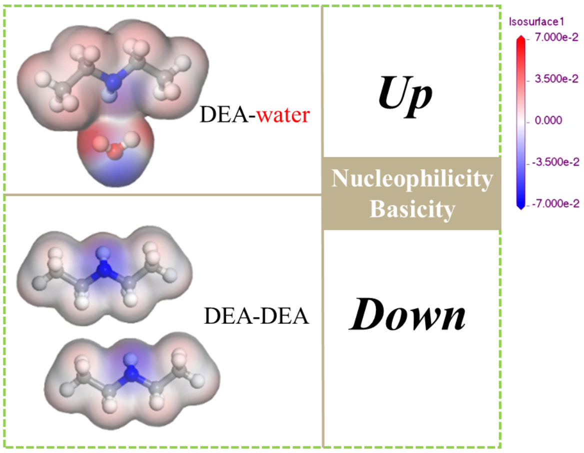

Supplementary Figure 3. The electrostatic potential map of DEA molecule with and without water.

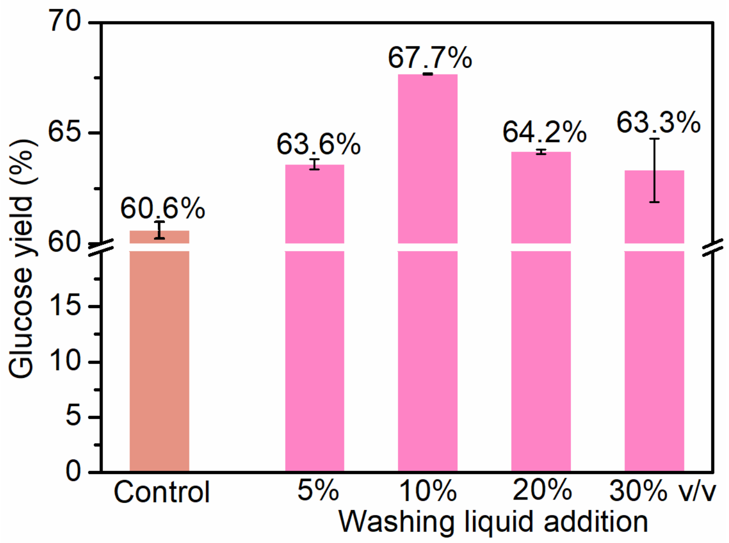

Supplementary Figure 4. The lignin-rich stream from DEA-based fractionation can be used as a promoter for the enzymatic hydrolysis. The stream was acidified to pH 7 prior to addition in the enzymatic hydrolysis system. Conditions in enzymatic saccharification: corn cob residue (CCR) as the substrate, 2% w/v of glucan loading, 3 FPU Celluclast 1.5L/g glucan, 5 CBU β-glucosidase/g glucan, 50 °C, pH 4.8, 150 rpm, and 72 h. Error bars represent the standard deviation.

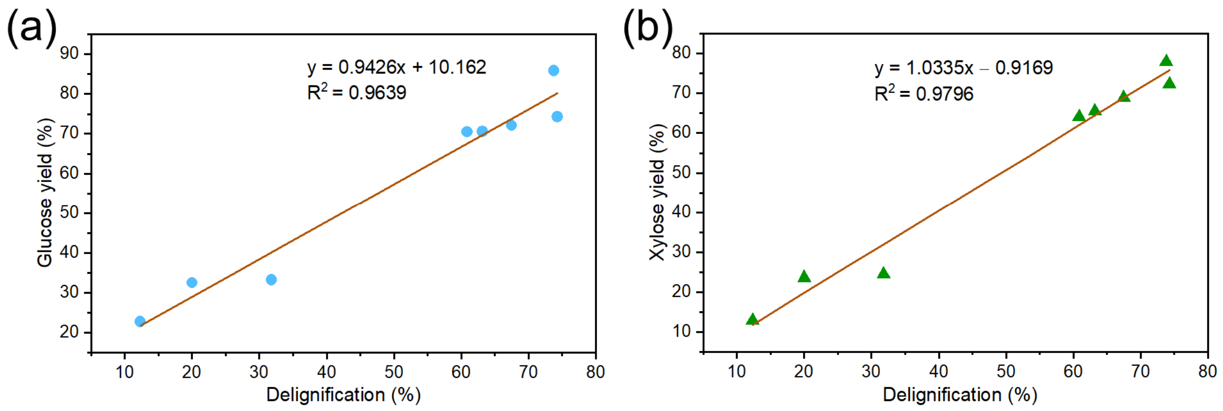

Supplementary Figure 5. Correlation between glucose (a) and xylose (b) yields and delignification.

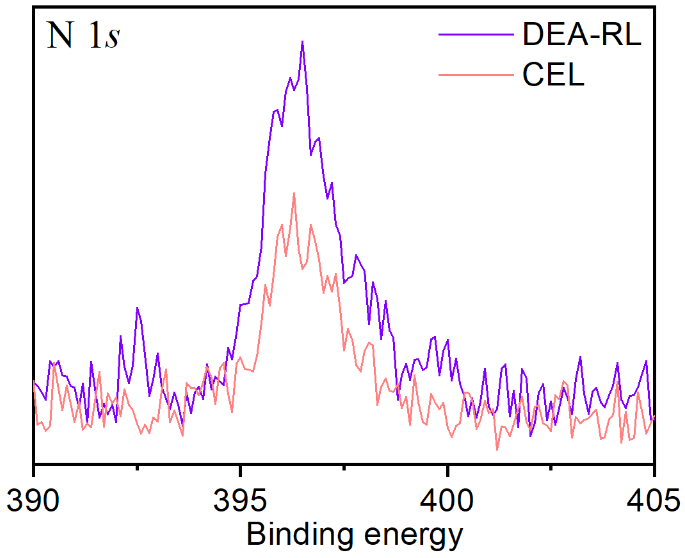

Supplementary Figure 6. XPS spectra of N 1*s* for DEA-RL and CEL.

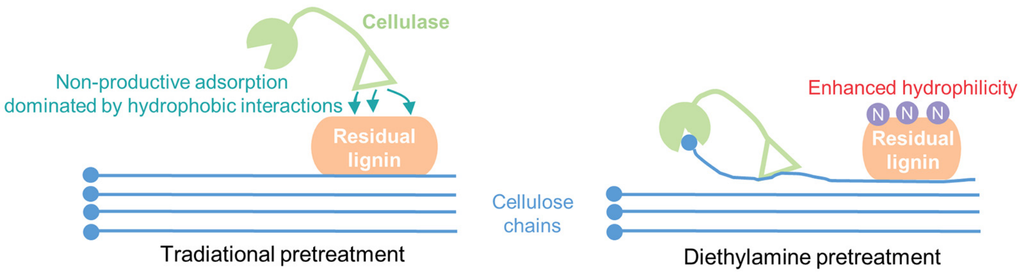

Supplementary Figure 7. Dissimilarity of adsorption behaviors of cellulase on residual lignins after traditional and DEA pretreatments.

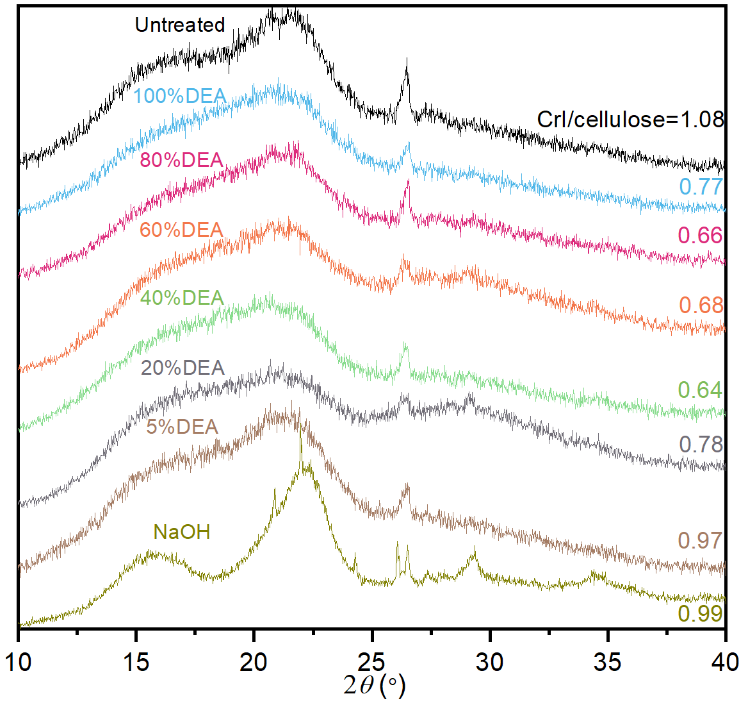

Supplementary Figure 8. XRD diffraction profiles of untreated and pretreated corn stover.

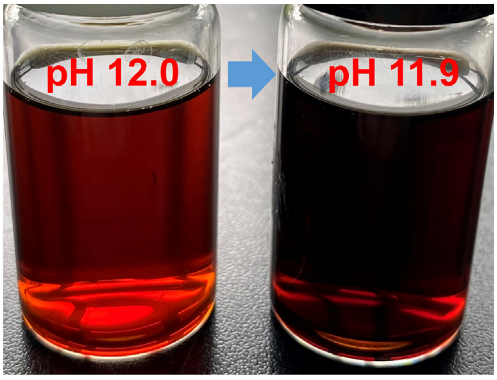

Supplementary Figure 9. Changes in the color of the NaOH pretreatment liquor before and after hydrogenolysis.

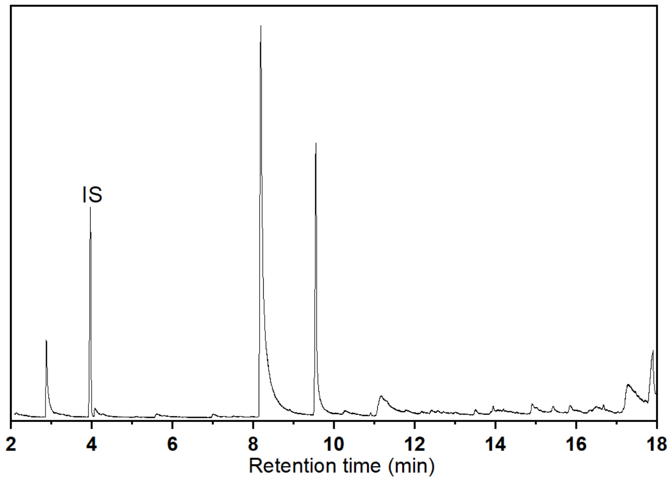

Supplementary Figure 10. GC-MS profile of the compounds in ethyl acetate extracts from the DEA pretreatment liquor before hydrogenolysis.

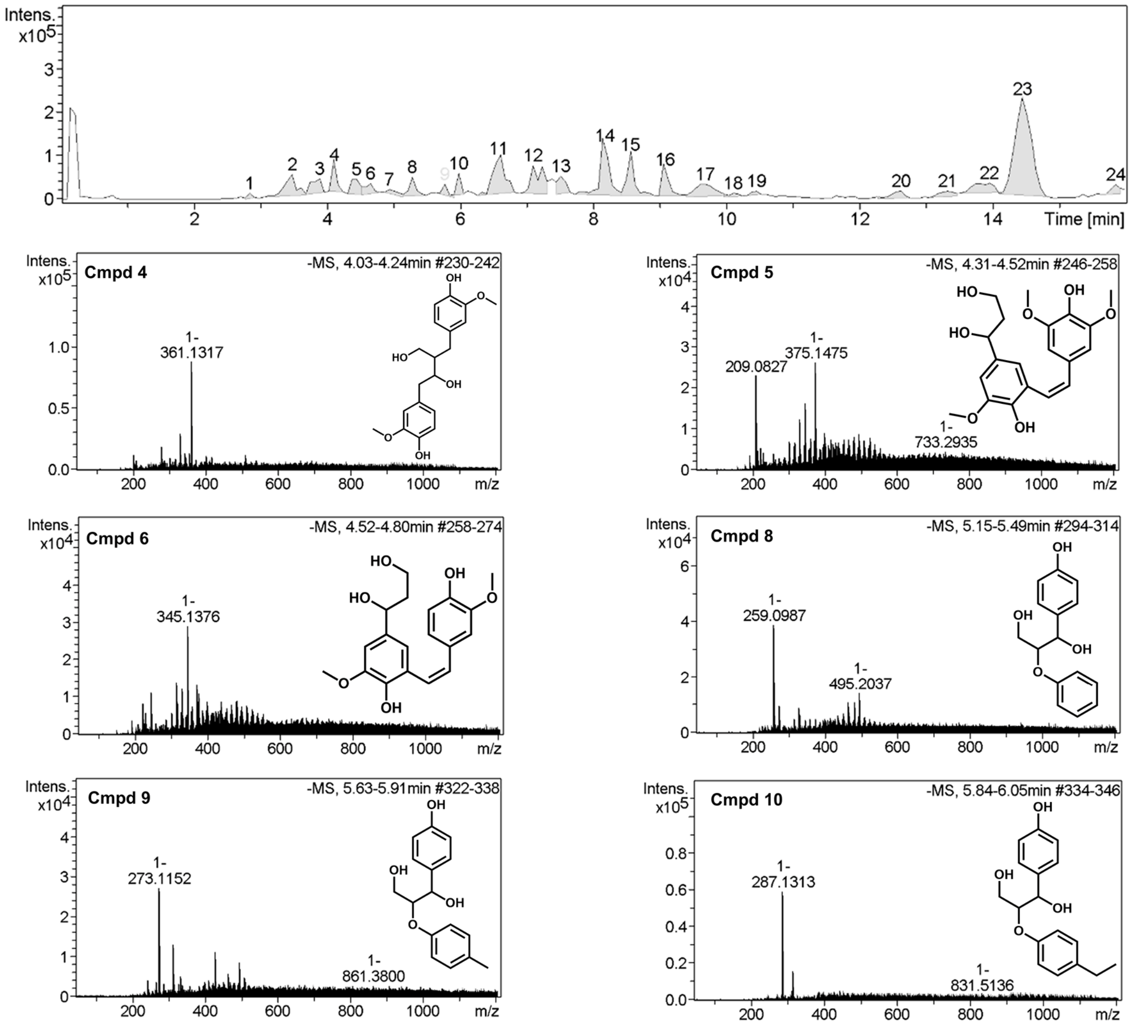

Supplementary Figure 11. LC-MS analysis of the products in ethyl acetate extracts from the DEA pretreatment liquor after hydrogenolysis.

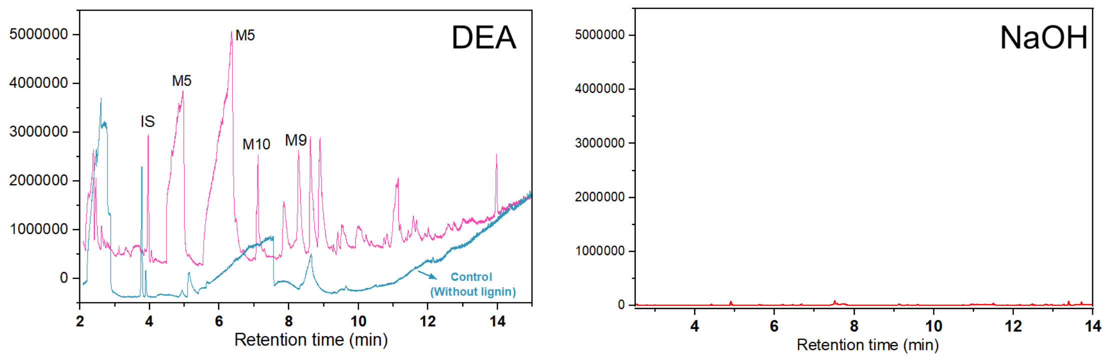

Supplementary Figure 12. GC-MS analysis of products in the aqueous phase after ethyl acetate extraction for the hydrogenolysis samples from the DEA and NaOH pretreatment liquors. Control group (18 bar H_2, 250 °C, 240 min, 400 rpm): Water (20 mL) + DEA (1 mL) + 10% Pd/C (50 mg) + copper acetate monohydrate (0.12 g).

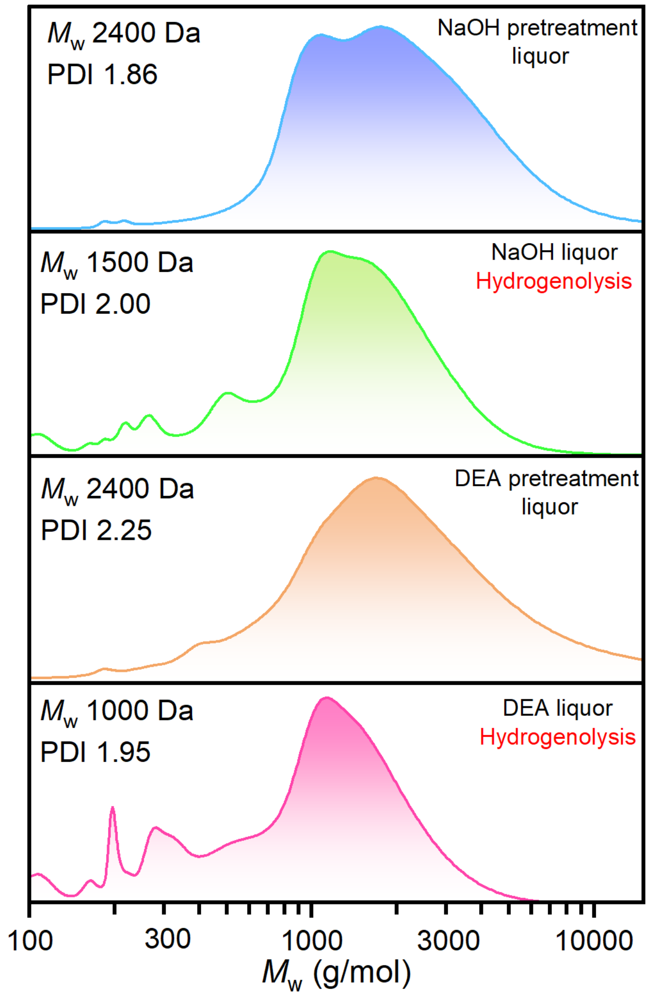

Supplementary Figure 13. GPC results of the lignins from DEA and NaOH pretreatment liquors before and after hydrogenolysis.

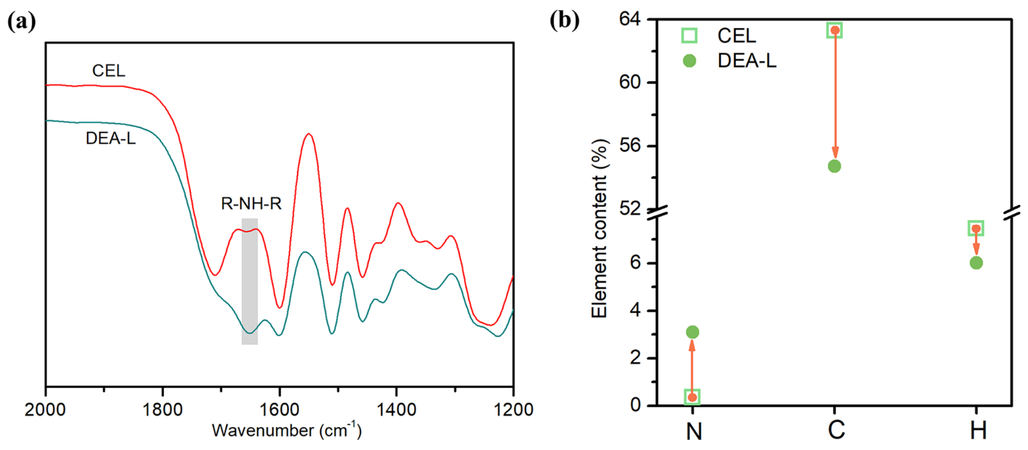

Supplementary Figure 14. FTIR spectra (a) and element analysis (b) of DEA-L and CEL.

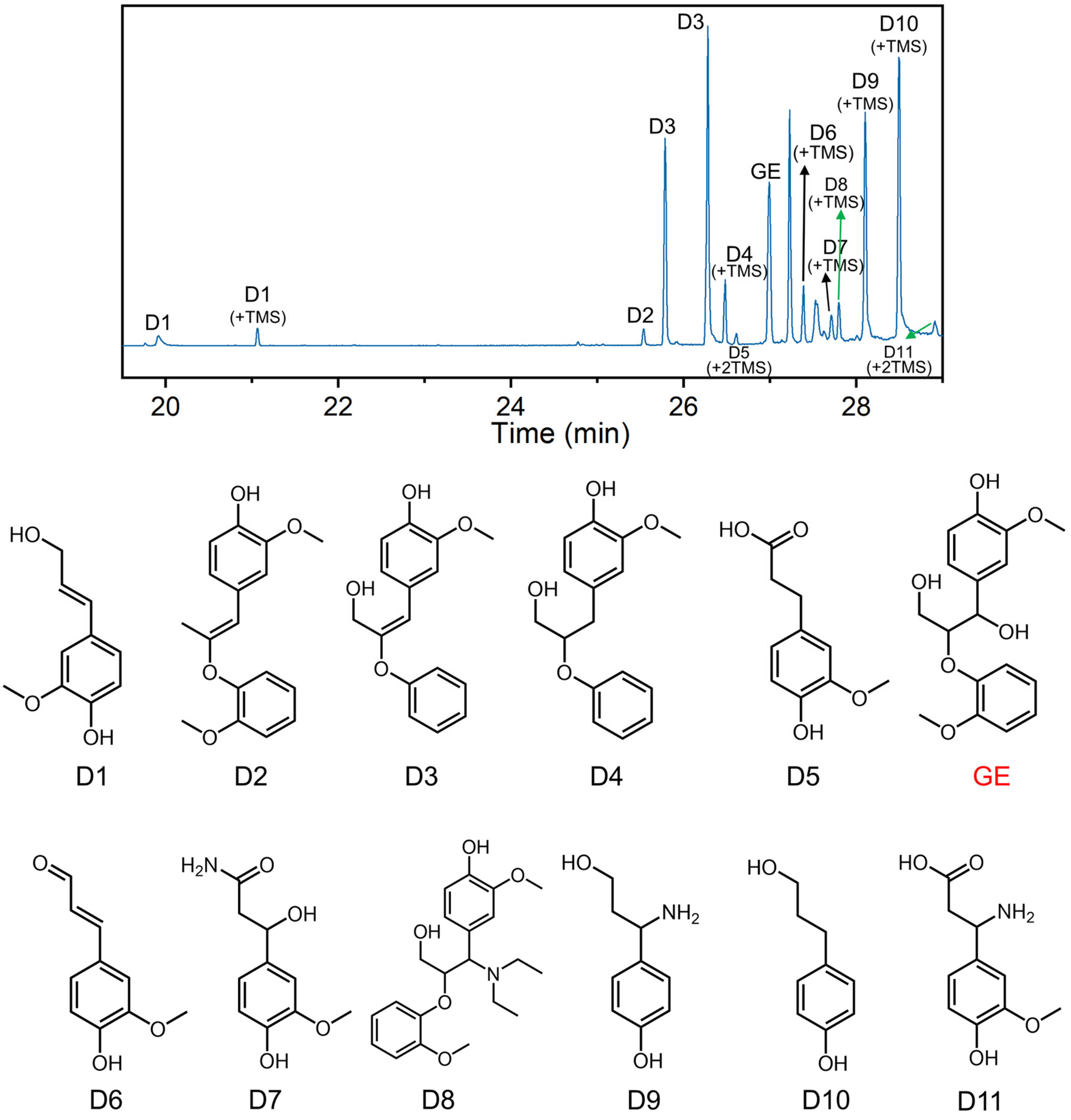

Supplementary Figure 15. The product distribution of the lignin model compound (GE) treated in an aqueous DEA system.

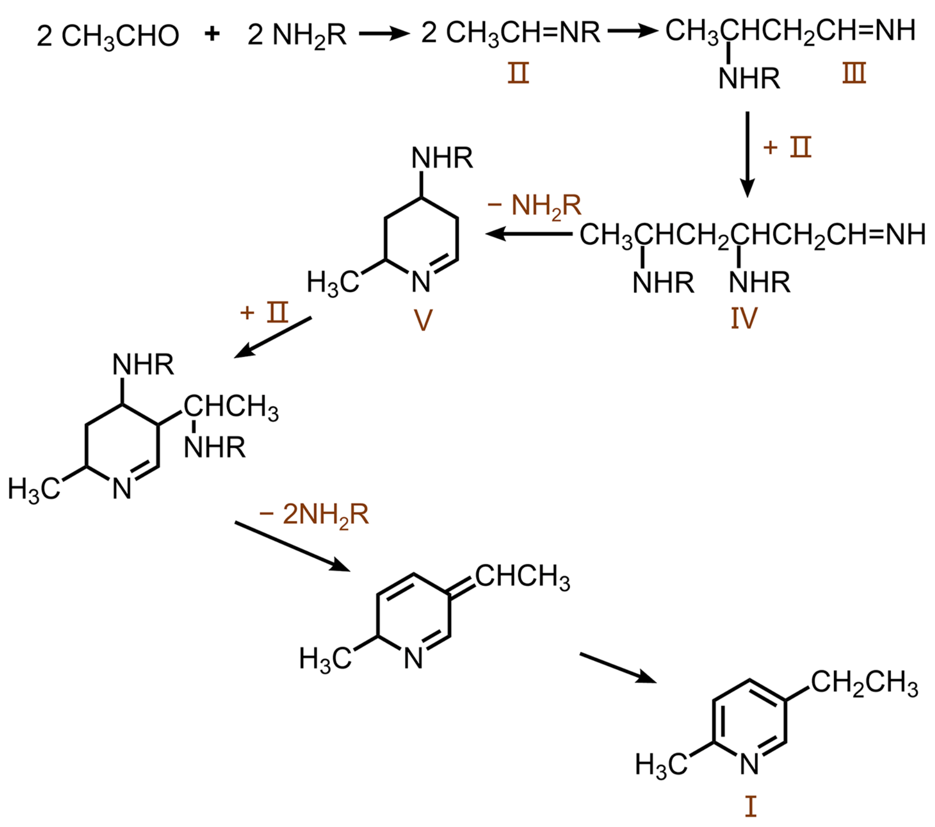

Supplementary Figure 16. Possible pathway to produce 5-ethyl-2-methylpyridine from the reaction between lignin-derived aldehydes and amines/ammonia. I: 5-Ethyl-2-methylpyridine; II: Aldimine; III: Amino imine/Amine imine; IV: Diamino imine/Diamine imine; V: Tetrahydropyridine.

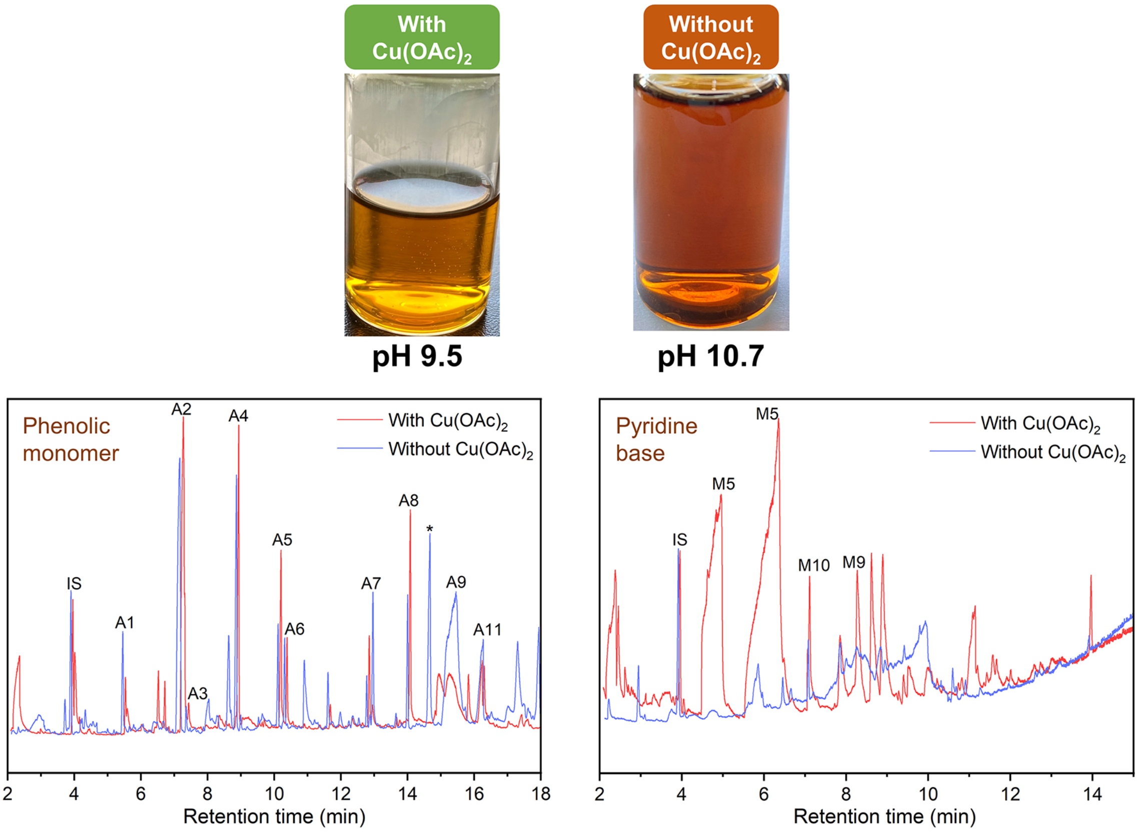

Supplementary Figure 17. Comparison of the hydrogenolysis products with and without $Cu(OAc)_2$. The structures of phenolic monomer (**A1**–**A11**) and substituted pyridine (**M5**, **M9**, and **M10**) compounds refer to Fig. 2. *: 4-Propanolguaiacol.

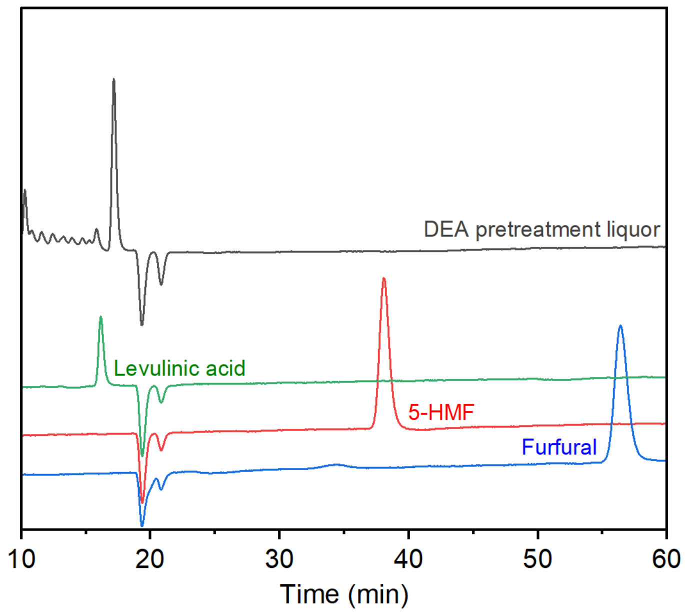

Supplementary Figure 18. HPLC profiles of the DEA pretreatment liquor, levulinic acid, 5-HMF, and furfural.

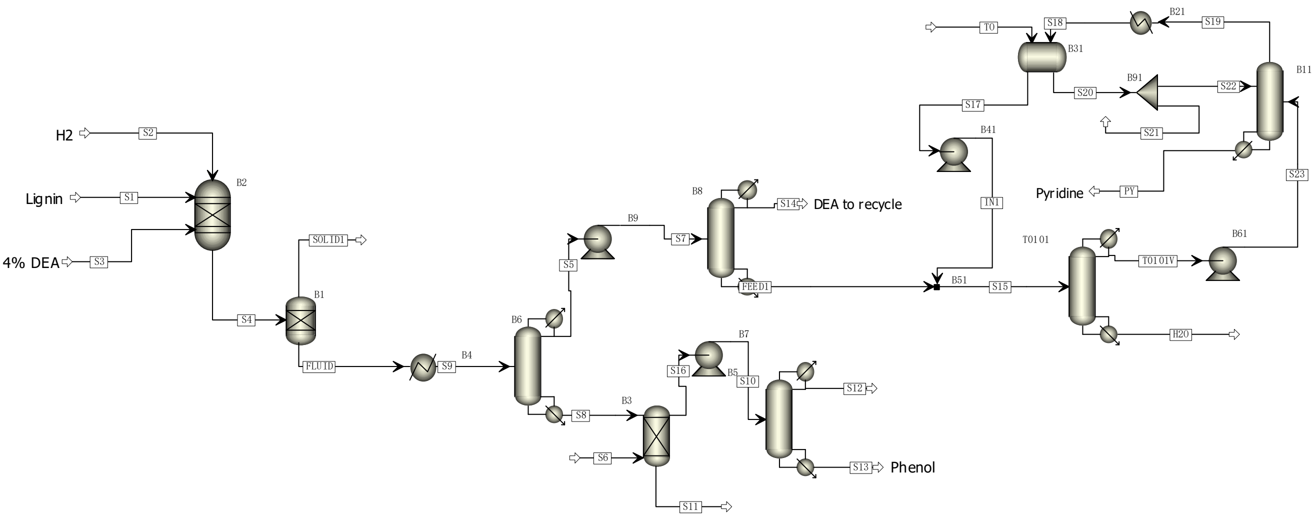

Supplementary Figure 19. Aspen Plus process flow diagram of the N-participated lignin valorization to pyridine bases and phenols. The separation and recycling were as follows: The outlet material from the reactor is directed into the primary separation tower (B6). From the primary separation tower kettle, phenolic wastewater is discharged. Following a 5-stage extraction process using methyl isobutyl ketone solvent (B3), the phenolic solvent is directed to the solvent recovery tower for the extraction of crude phenol. The upper material from the primary separation tower contains a substantial amount of DEA and pyridine. It is then introduced into the DEA recovery tower (B8), where DEA is separated and recovered from the tower's top. The kettle discharge from the tower contains water and pyridine, which is subsequently fed into the azeotropic distillation unit, using *n*-propyl formate as the azeotrope agent. This process ultimately accomplishes the separation of water from pyridine.

4. Supplementary Tables

Supplementary Table 1. Gluco- and xylo-oligomer content in DEA pretreatment liquors.

DEA: water (v/v)	Gluco-oligomer content (%)[a]	Xylo-oligomer content (%)[b]
100: 0	-	4.5 ± 0.1
80: 20	-	17.0 ± 0.3
60: 40	2.0 ± 0.1	18.8 ± 0.3
40: 60	2.6 ± 0.1	19.5 ± 0.2
20: 80	1.9 ± 0.1	17.6 ± 0.3
5: 95	-	-

a: Gluco-oligomer content was calculated based on the ratio of gluco-oligomer weight in pretreatment liquor to glucan weight in starting corn stover.

b: Xylo-oligomer content was calculated based on the ratio of xylo-oligomer weight in pretreatment liquor to glucan weight in starting corn stover.

Supplementary Table 2. Financial assumptions and design basis.[4]

Case	Value
Plant life	30 years
Cost year (dollar unit)	2014 dollars
Capacity factor	90%
Discount rate	10%
General plant depreciation	200% declining balance
General plant recovery period	7 years
Financing	40% equity
Loan terms	10-year loan at 8% annual percentage rate
Construction period	3 years
First 12 months' expenditures	8%
Next 12 months' expenditures	60%
Last 12 months' expenditures	32%
Working capital	5% of fixed capital investment
Start-up time	3 months
Revenues during start-up	50%
Variable costs during start-up	75%
Fixed costs during start-up	100%
Lignin ($/ton)	55.8
Diethylamine ($/ton)	1500

Supplementary Table 3. Mass flow of key streams.

Component	Units	S1	S3	S4	S13	S7	S14	S15	PY
Lignin	kg/hr	700	-	454.983	-	-	-	-	-
DEA	kg/hr	-	784.819	685.436	-	685.362	675.98	31.962	-
Pyridine	kg/hr	-	-	139.995	0.00047	139.505	0.05946	139.566	139.488
Phenol	kg/hr	-	-	104.996	102.005	2.94737	-	2.97247	1.14177
H_2O	kg/hr	-	18835.6	18914.2	-	377.299	6.91979	470.898	-

Supplementary Table 4. Techno-economic analysis (TEA) of the lignin valorization to pyridine bases and phenols.

	Value
Annual production/MMkg	1.94
Total capital cost/MM$	31.65
Total operation cost/MM$/yr	2.1
Raw material/MM$/yr	1.5
Utilities/MM$/yr	0.54
Rate of return/%	10
Minimum pyridine selling price/$/kg*	2.8

*MPSP was calculated based on the coproduction of phenols with a selling price of $1.3/kg.

References

1. Ghose, T. K. Measurement of cellulase activities. *Pure Appl. Chem.* **59**, 257-268 (1987).
2. Ai, B. et al. Natural deep eutectic solvent mediated extrusion for continuous high-solid pretreatment of lignocellulosic biomass. *Green Chem.* **22**, 6372-6383 (2020).
3. Dao, T. H. et al. Identification and quantification of lignin monomers and oligomers from reductive catalytic fractionation of pine wood with GC × GC – FID/MS. *Green Chem.* **24**, 191-206 (2022).
4. Liu, Z. H. et al. Transforming biorefinery designs with 'Plug-In Processes of Lignin' to enable economic waste valorization. *Nat. Commun.* **12**, 3912 (2021).
5. Shen, R., Tao, L. & Yang, B. Techno-economic analysis of jet-fuel production from biorefinery waste lignin. *Biofuels, Bioprod. Biorefin.* **13**, 486-501 (2019).
6. Humbird, D. et al. Process Design and Economics for Biochemical Conversion of Lignocellulosic Biomass to Ethanol: Dilute-Acid Pretreatment and Enzymatic Hydrolysis of Corn Stover. National Renewable Energy Lab. (NREL), Golden, CO (United States) (2011).
7. Yamamura, M., Hattori, T., Suzuki, S., Shibata, D. & Umezawa, T. Microscale alkaline nitrobenzene oxidation method for high-throughput determination of lignin aromatic components. *Plant Biotechnol.* **27**, 305-310 (2010).
8. Fan, D. et al. Microwave-assisted fractionation of poplar sawdust into high-yield noncondensed lignin and carbohydrates in methanol/p-toluenesulfonic acid. *Chem. Eng. J.* **454**, 140237 (2023).
9. Xu, L. et al. Unveiling the role of long-range and short-range forces in the non-productive adsorption between lignin and cellulases at different temperatures. *J. Colloid Interf. Sci.* **647**, 318-330 (2023).
10. Parthasarathi, R., Romero, R. A., Redondo, A. & Gnanakaran, S. Theoretical Study of the Remarkably Diverse Linkages in Lignin. *J. Phys. Chem. Lett.* **2**, 2660-2666 (2011).
